# Performance of the Front-of-Pack Nutrition Label Nutri-Score to Discriminate the Nutritional Quality of Foods Products: A Comparative Study across 8 European Countries

**DOI:** 10.3390/nu12051303

**Published:** 2020-05-02

**Authors:** Louise Dréano-Trécant, Manon Egnell, Serge Hercberg, Pilar Galan, Juliette Soudon, Morgane Fialon, Mathilde Touvier, Emmanuelle Kesse-Guyot, Chantal Julia

**Affiliations:** 1 Nutritional Epidemiology Research Team (EREN), Epidemiology and Statistics Research Center, University of Paris (CRESS), Sorbonne Paris Nord University, Inserm U1153, Inrae U1125, Cnam, 93017 Bobigny, France; louise.dreanotrecant@gmail.com (L.D.-T.); s.hercberg@eren.smbh.univ-paris13.fr (S.H.); p.galan@eren.smbh.univ-paris13.fr (P.G.); juliette.soudon@gmail.com (J.S.); m.fialon@eren.smbh.univ-paris13.fr (M.F.); m.touvier@eren.smbh.univ-paris13.fr (M.T.); e.kesse@eren.smbh.univ-paris13.fr (E.K.-G.); c.julia@eren.smbh.univ-paris13.fr (C.J.); 2 Public Health Department, Avicenne Hospital, Assistance Publique des Hôpitaux de Paris (AP-HP), 93000 Bobigny, France

**Keywords:** front-of-pack nutritional labelling, European countries, discriminating performance, nutritional recommendations

## Abstract

In Europe, discussions are currently ongoing to harmonize front-of-pack nutritional labelling, while some countries have adopted or are considering implementing the Nutri-Score. However, its adaptability to multiple nutritional contexts in Europe requires further investigation. This study aimed to evaluate the applicability of the Nutri-Score in various European countries, regarding its ability to discriminate the nutritional quality of foods and its consistency with national dietary recommendations. The European Food Information Resource (EUROFIR) nutritional composition databases from eight European countries (Finland, France, Norway, Poland, Portugal, Slovakia, Sweden, and Switzerland) were used. The distribution of foods across the Nutri-Score classes within food groups was assessed, as well as the discriminating performance of the label, and the adequacy of nutritional recommendations. The Nutri-Score demonstrated high discriminating ability for all food groups, with similar trends in the eight countries, and consistency with nutritional recommendations. For instance, fruit and vegetable products were mainly classified in the two healthiest Nutri-Score categories, while sugar and animal fat products were mainly classified in the two less healthy categories of the Nutri-Score. Our results support the fact that the Nutri-Score would be a relevant tool to discriminate the nutritional quality of products within and across relevant food groups in different European countries, with consistency with nutritional recommendations.

## 1. Introduction

In 2017, the European region was the most affected World Health Organization (WHO) region by non-communicable diseases (e.g., diabetes, cardiovascular diseases, cancer, chronic respiratory diseases and mental disorders), accounting for approximately 77% of the burden of disease and 88% of deaths [1]. Nutrition and physical activity have rapidly become major thrusts of public health action [2]. Despite nutritional recommendations and the various health actions taken by public authorities at national, European and international levels, the mortality rate and prevalence of these diseases continue to rise [3]. In this context, new public health approaches have been set up, including notably the implementation of nutritional information systems on the front of pack of food products. In recent years, the implementation of front-of-pack nutrition labels (FoPLs) has been identified to be of major interest by expert committees in charge of nutrition labelling policies in many countries, by the WHO [4] and the Organization for Economic Co-operation and Development [5]. Indeed, FoPLs aim to improve the nutritional status of populations, both by encouraging consumers to make healthier food choices at the point of purchase [6,7,8], and by enticing manufacturers to improve the nutritional quality of food offered [9,10].

However, in view of the plurality of FoPLs on the European market, potentially leading to confusion for consumers, the European Commission has launched discussions among member states on the potential reopening of the INCO regulation [11], including a reflection on the harmonization of FoPLs [12]. Several schemes of FoPLs have been developed worldwide and notably in Europe, including nutrient-specific systems providing information at the nutrient level (e.g., the multiple traffic lights implemented in the United Kingdom since 2004 [13], or the reference intakes adopted by multiple European food manufacturers in 2006 [14] or their variants such as the Italian battery system [15]) and summary indicators synthetizing the overall nutritional quality of foods (e.g., the Keyhole in Scandinavian countries [16] or a graded scale such as the Nutri-Score, first adopted in France in 2017 [17], then in Belgium, Spain, Germany, the Netherlands, Switzerland and Luxembourg between 2018 and 2020).

More specifically, the Nutri-Score is a color-coded graded scale of five letters based on the United Kingdom’s Food Standards Agency (FSA) nutrient profiling system (NPS), adapted to the French context by the French High Council for Public Health (HCSP), namely the FSAm-NPS [18,19]. The validation of a FoPL requires validating the graphical format of the system as well as its underlying NPS [20,21]. Numerous studies in the French context, and one study in other countries including some European countries, have been carried out to validate the graphical format of the Nutri-Score, regarding several dimensions of its effectiveness. First, the Nutri-Score has been shown to be well perceived and understood by consumers to compare the nutritional quality of foods [22,23,24,25,26,27], two prerequisites for the FoPL use. Further, this FoPL has also been demonstrated to encourage consumers towards healthier food choices and improve the nutritional quality of their shopping carts [28,29,30,31], and finally to potentially decrease the mortality from nutrition-related chronic diseases through healthier dietary intakes, according to a simulation study [32]. Regarding the FSAm-NPS underpinning the Nutri-Score, several validation studies testing the Nutri-Score discriminating performance against food composition have been conducted in particular in the French context, and have notably demonstrated its capacity to discriminate the nutritional quality of food products across and within food groups [33,34], and its consistency with French nutritional recommendations [33]. A scientific report has also suggested consistent results in some European countries [35,36]. However, no formal study has yet been conducted to investigate the discriminating capacity of the Nutri-Score in several European countries, while it constitutes an important aspect of its transferability to other food contexts and that a growing number of European countries are considering its implementation. Thus, the present study aimed to investigate the discriminating performance of the Nutri-Score in terms of the food offered in different European countries, as well as the consistency of the classification with nutritional recommendations of these countries.

## 2. Materials and Methods 

### 2.1. Food Composition and Classification 

Data were retrieved from the European Food Information Resource (EUROFIR), an international non-profit association created to promote international cooperation in the nutrition field, in particular through the collection of validated national nutritional databases [37]. Following a licensing agreement, EUROFIR provided us with access to food classification and composition tables of common and specific foods from different European countries.

#### 2.1.1. Food Classification

Food products were categorized according to the EUROFIR classification, grouping foods with similar use and nutritional characteristics (Supplemental Material 1). Herbs and spices, alcoholic beverages, dietary supplements and foods for special nutritional use were excluded as they are not included in the scope of the Nutri-Score according to the European regulation [19]. Each food was exclusively classified into a single main group and subgroup. the main food groups were as follows: “egg or egg product”, “fat or oil”, “fruit or fruit product”, “grain or grain product”, “meat or meat product”, “milk, milk product or milk substitute”, “composite food product”, “nut, seed or kernel”, “seafood or related product”, “sugar or sugar product”, “vegetable or vegetable product”, and “beverage non-milk”. Within each main food group, subgroups were assigned using also the harmonized EUROFIR classification (e.g., the main group “grain or grain product” included “bread and similar products”, “breakfast cereals and cereal bars”, “cereal or cereal-like milling products and derivatives”, “fine bakery ware”, “and pasta, rice and other cereals” as subgroups). In total, 12 main food groups and 41 subgroups were defined. 

#### 2.1.2. Food Composition

The following elements in the nutritional composition table of each country were required for the calculation of the FSAm-NPS score and thus the Nutri-Score of each food: energy, sugars, fats, Saturated Fatty Acids (SFA), proteins, fiber, and sodium. In order to limit the number of missing data, the content of some nutrients was recalculated using usual equations with other data available in the tables (e.g., total energy (kJ) = total protein (g) * 17 + total carbohydrates (g) * 17 + total fat (g) * 38 + alcohol (g) * 29 + total dietary fiber (g) * 8). Moreover, in order to verify the quality of the nutritional composition data, control procedures were performed on variables of interest for the FSAm-NPS calculation, including verification on the total energy content. The analyses were finally carried out on the eight following countries, for which all necessary data were available: Finland, France, Norway, Poland, Portugal, Slovakia, Sweden, and Switzerland. Finally, 11,347 foods and beverages from the eight countries contained data on food composition and classification and were thus used for the analyses. The number of food products available in each group or subgroup varied across countries. In total, 2075 food products were included in the database for Finland, 2309 for France, 1101 for Norway, 919 for Poland, 921 for Portugal, 1183 for Slovakia, 1990 for Sweden and 849 for Switzerland. The flowchart of the food databases used in the present study is described in Supplemental Material 2.

### 2.2. Nutri-Score Computation

Based on the nutritional composition for 100 g of food (or 100 mL of beverage), the FSAm-NPS assigns positive points for the amounts in elements that should be limited, including energy (kJ), total sugars (g), SFA (g), and sodium (mg) (0 to 10 positive points for each). Negative points are then attributed to the amount in elements that should be promoted, including fruits, vegetables, legumes and nuts (FVLN, %), fibers (g) and protein (g) (0 to 5 negative points for each). The FVLN component was not available per se in the composition tables. Thus, values were estimated for each product, using French generic food composition databases for which FVLN amounts had been estimated [33,35,38,39]. The estimate of the percentage of FVLN for composite dishes was verified by trained dieticians. By summing the negative points to the positive points, an overall discrete score was then obtained ranging from -15 (for products with higher nutritional quality) to +40 points (for products with lower nutritional quality). Specific modifications were applied to beverages, cheeses, fats and oils, according to the adaptations of the French HCSP [40]. Finally, thresholds were applied to compute the Nutri-Score of each food: “A” below –1 point (in dark green), “B” from 0 to 2 points (light green), “C” from 3 to 10 points (yellow), “D” from 11 to 18 points (light orange), and “E” from 19 points and more (dark orange). For beverages, the thresholds were the following: “A” applies only to water (dark green), “B” up to 1 point (light green), “C” from 2 to 5 points (yellow), “D” from 6 to 9 points (light orange), and “E” above 10 points (dark orange). The algorithm to calculate the FSAm-NPS score has been described elsewhere [40]. 

### 2.3. Statistical Analyses

For each main food group and subgroup, the distribution of foods and beverages in the different classes of the Nutri-Score was computed (i.e., the number of foods per Nutri-Score class and the corresponding percentage). This distribution was assessed across and within the eight countries. Further, within each main food group and subgroup, the modal class and the percentage corresponding to the predominant Nutri-Score class were calculated. The ability of the Nutri-Score to discriminate the nutritional quality of products within main food groups and subgroups was assessed using the number of available colors in each group. Discriminating performance was considered satisfying when at least three classes of Nutri-Score were available in the food group. In addition, the overall distribution of foods in the different classes of the Nutri-Score was compared with the food-based dietary guidelines from the different countries included in the study and the general guidelines from the WHO [41]. Thus, food groups for which consumption is encouraged by recommendations should be mainly classified as higher nutritional quality by the Nutri-Score (“A” or “B”), while groups for which consumption has to be limited should be mainly classified as lower nutritional quality (“D” or “E”).

## 3. Results

Overall, a final sample of 11,347 foods and beverages was used, including 1800 fresh or processed fruits, vegetables and nuts, seeds or kernels; 1898 breads and cereal products; 2506 meat, fish and egg products; 906 milk and dairy products; 336 fats or oils; 2655 composite products; 614 sugar or sugar products; and 632 beverages.

**Figure 1:**
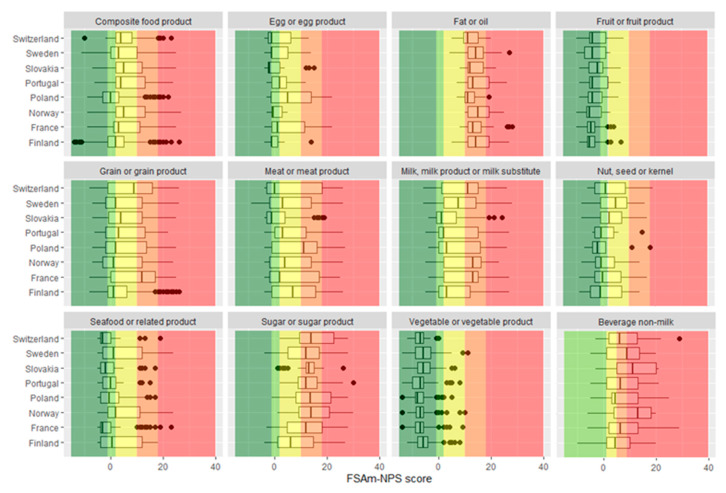
Overall distribution of products within the main food groups and across countries. Dark green: Nutri-Score “A”, light green: Nutri-Score “B”, yellow: Nutri-Score “C”, light orange: Nutri-Score “D”, and dark orange: Nutri-Score “E”. No Nutri-Score “A” was represented on the graphic of beverages, given that only waters can be classified as “A” and were thus excluded from the graphic. Values are the number of food products (%).

Overall, for the eight countries combined, the distribution of the different Nutri-Score classes for each main food group and subgroup is displayed in Table 1, and results by country are described in the tables from Supplemental Material 3 and Figure 1. The distribution of the various main groups and subgroups within the Nutri-Score classes was globally consistent with dietary guidelines [41], overall and for each of the eight countries. Indeed, 96.4% of foods from “vegetable or vegetable product” and 91.1% of foods from “fruit or fruit product” were classified in the two healthiest Nutri-Score classes (“A” and “B”). In addition, 55.4% of the products from “grain or grain product” and 71.2% of the products from “nut, seed or kernel” were generally distributed between classes “A” to “C”. In addition, more than three-quarters of the products from “fish or related organisms” (88.4%) and “pulse or pulse product” (100%) were classified into the first two classes of Nutri-Score (“A” and “B”). On the contrary, 87.8% of the products from “sugar or sugar product” were classified between “C” and “E”, and 82.4% of the products from “fat or oil” were classified in the categories “D” and “E” of the Nutri-Score (i.e., to be limited), with a better ranking for vegetable fats (24.8% in “C”, 66.4% in “D”, and 8.9% in “E”) compared to animal fats (1.6% in “C”, 68.8% in “D”, and 18.8% in “E”). Similar trends were observed in the analyses by country (Figure 1, Supplemental Material 3). Indeed, for example, for the “fruit or fruit product” category, 97.3% were classified as “A” or “B” with the Nutri-Score in Finland, 95.9% in France, 97.1% in Norway, 92.6% in Poland, 77.8% in Portugal, 87.5% in Slovakia, 89.3% in Sweden, and 91.4% in Switzerland. For the “sugar and sugar product” category, 66.1% of products were classified “C”, “D” or “E” with the Nutri-Score in Finland, 88.3% in France, 96.3% in Norway, 98.0% in Poland, 97.6% in Portugal, 97.2% in Slovakia, 88.3% in Sweden, and 97.4% in Switzerland. Only a few disparities were found between countries regarding the variability in the FSAm-NPS score within some of the main food groups (e.g., “seafood and related product”, “composite food product”). Nevertheless, for the main food groups “meat or meat product” and “milk, milk product or milk substitute”, differences were observed across countries regarding the distribution of the FSAm-NPS score of products, which may be partly explained by the large diversity of products in these food categories.

Overall, in the eight countries combined, at least three classes of the Nutri-Score were observed for all main groups and subgroups, except in the “pulse and pulse product” subgroup, where only two classes of the Nutri-Score (“A” and “B”) were represented. The results on the overall discriminating performance of the Nutri-Score in each country are displayed in Table 2. For each main group and subgroup, differences in the nutritional quality of products were grasped by the Nutri-Score classification, with a good discriminating performance in the eight countries. Indeed, for each country, 70% to 88% of the subgroups (excluding “Water”) contained food products categorized in at least three classes of the Nutri-Score, except for Poland (55%) and Switzerland (59%), containing fewer food products overall. In addition, the classification with the Nutri-Score allowed discriminating the wide variability in the nutritional quality of manufactured foods. Indeed, in the category “grain or grain product” for example, from 81% to 100% (depending on the country) of pasta and rice products were mainly identified in categories “A” and “B”, while more than half of the subgroup “breakfast cereal”, which are considered as highly processed manufactured foods, were classified in categories “C” and “D” for most of the countries. Within food categories, the Nutri-Score seemed to discriminate between refined and whole products. Indeed, for example, wholegrain breads were better classified than white breads.

## 4. Discussion 

In this study, the distribution of foods within the Nutri-Score classes showed a good performance of the FoPL to discriminate the nutritional quality of products within main food groups and subgroups, and across relevant food groups in terms of purchase, use or consumption, with high consistency with official dietary guidelines in the eight countries tested—Finland, France, Norway, Poland, Portugal, Slovakia, Sweden and Switzerland. 

These results are consistent with other studies using food composition databases of manufactured foods from the French and German markets, therefore validating the ability of the Nutri-Score to discriminate the nutritional quality of products in various sociocultural contexts [33,35]. A scientific report has also suggested a good discriminating performance of the Nutri-Score for the food offered in Belgium, Italy, Netherlands, Spain, Sweden, Switzerland and United Kingdom, using the data from the Open Food Facts database, a collaborative online project gathering food composition data on manufactured foods from many countries worldwide [36]. Furthermore, our findings showed good consistency between the classification of the Nutri-Score and the general recommendations of dietary guidelines [41]. Indeed, the vast majority of fruit, vegetable, legume and nut products for which consumption is encouraged had a better ranking with the Nutri-Score (mainly “A” and “B”) compared to sweet, fatty or salty products for which consumption should be limited (mainly “D” and “E”). The composite dishes showed a wide distribution (often 4 or 5 Nutri-Score classes represented in the food subgroups), which highlighted the large variability in prepared products in terms of nutritional quality, for which the Nutri-Score is a very useful tool to identify healthier options. Beverages such as water and unsweetened drinks were classified as healthier than sweetened drinks such as soft drinks and fruit nectars. All these findings are therefore in line with international key messages encouraging the consumption of fruits and vegetables, basic starches and whole cereals products, and limiting the consumption of fats, salt and sugar [4,41,42,43]. Our results also highlighted the fact that the Nutri-Score could allow discriminate between manufactured and raw foods. Indeed, processed foods from the various European countries were mainly distributed in classes “C”, “D”, or “E”, while raw products were usually classified in “A” or “B”, which is consistent with new public health messages encouraging the consumption of these products [41]. Moreover, the Nutri-Score appeared to allow discrimination between refined and whole foods. For example, in most European countries included in the study, wholemeal bread was classified as better with the Nutri-Score than white bread. Finally, the Nutri-Score also seemed to be consistent for products specific to a food context for which consumption is encouraged. This was notably the case for rye breads—rich in fibers—specific to Nordic countries and classified as “A” with the Nutri-Score, while key messages encourage diet oriented towards the consumption of whole seeds [44]. Thus, our findings suggest good consistency between food classification by the Nutri-Score and overall as well as some specific nutritional recommendations at the European level, in line with previous studies that have been carried out [33,35,41].

The validation of the ability of the Nutri-Score to discriminate the nutritional quality of products in different markets using the FSAm-NPS profile is particularly important to assess its transferability given the current European context. Beyond the country-specific food database, the concordance of our results confirms the performance of the Nutri-Score system in achieving product discrimination in various European countries [20,21]. Only a few disparities were found between countries regarding the variability or the distribution of the FSAm-NPS score within some of the main food groups, which might be partly explained by the structure of the original food databases and the specific food offered and contexts of the different countries included in the study. Nevertheless, it is important to notice that even if some disparities can be observed regarding the classification of foods by a front-of-pack nutritional labelling system in various food environments, it does not necessarily imply that adaptations of the underlying profiling system are required, as disparities in the distribution of foods are not necessarily inconsistencies. These findings are complementary to the results of an international comparative study, which found that the Nutri-Score was an effective tool to help consumers identify the nutritional quality of products and potentially improve their food choices in multiple countries, including European countries—Bulgaria, Denmark, France, Germany, the Netherlands, Spain, and the United Kingdom [26,31,45]. 

Dietary guidelines and FoPLs such as the Nutri-Score are complementary and synergic measures, based on different approaches and principles. Dietary guidelines aim to provide consumers practical guidelines to adopt a healthy diet, by helping them identifying the food groups that should be encouraged (e.g., fruits, vegetables, legumes, etc.) and those that should be limited (e.g., sweet and salty products, fats and especially animal fats, etc.) [41]. Nevertheless, the consumption of manufactured foods has increased substantially [46], and the food offered currently, notably in Europe, are characterized by a large variability in nutrient profiles of pre-packed foods within food groups. Thus, to allow consumers to identify healthier products within a main food group or subgroup, the Nutri-Score appears to be an efficient tool to help consumers discriminate nutritional quality across and within food groups in multiple European countries, as well as between similar products from different brands in French supermarkets [33]. So, the Nutri-Score provides supplementary information to guide consumers toward foods with a better nutritional composition (with less unfavorable or more favorable elements). Nevertheless, consistent communication and specific education strategies are needed in each country to explain the complementarity and the synergic use of dietary guidelines and the Nutri-Score. 

The use of the EUROFIR database, including both generic and specific food and beverages of multiple European countries, is the main strength of the present study. Indeed, it allowed us to perform cross-cultural comparisons of the discriminating performance of the Nutri-Score, particularly important in the current European context of front-of-pack nutritional labelling harmonization. This comparative study across eight European countries remains the first study in terms of the validation of the NPS underlying the Nutri-Score in different sociocultural contexts on a large variety of generic foods. These findings are complementary to studies conducted in other food databases such as Open Food Facts providing data on multiple industrial foods of different brands. However, some limitations should be acknowledged. First, data from EUROFIR were retrieved from different sources (e.g., universities, research institutes, food technology institutes, food quality organizations and commercial organizations) and did not allow us to analyze the representativeness of the sample of foods collected in our database. Nevertheless, our objective was to assess the discrimination capacity of the Nutri-Score; we did not need to be exhaustive and the EUROFIR database provided a large number of food products for each country. In addition, some disparities were observed between countries regarding the number of foods available, or even the composition of foods or both. Given these heterogeneities of the database structures, we elected not to perform statistical tests to determine the significance of the intercountry differences. However, only a few countries (Poland, Portugal, and Switzerland) were concerned, and their results remain consistent with the literature mentioned above. Moreover, several procedures were applied to verify the validity of the data and optimize the number of products in each database. In addition, results on the distribution of foods in the different Nutri-Score classes depended on the EUROFIR classification that was used in the present study. Another limitation of the present study remains the somewhat arbitrary aspect of the measurements of the discriminating performance of the FoPL and the consistency with dietary guidelines. Nevertheless, in the absence of a consensual indicator to which to compare the performance of nutrient profiling, we used a similar methodology to previous studies [33,35]. Finally, given the current European context, the present study focused on the Nutri-Score discriminating performance in multiple European countries. Nevertheless, it would be interesting to replicate this type of study to investigate and compare the discriminating performance of various FoPL schemes implemented worldwide.

Our results provide additional evidence of the relevant application of the Nutri-Score and its adaptability to the European context, particularly in Finland, France, Norway, Poland, Portugal, Slovakia, Sweden, and Switzerland, with regard to discrimination within main food groups and subgroups. This FoPL is notably strongly supported by European consumer associations who launched a petition (“PRO-NUTRISCORE”) in order to encourage the European Commission to change the regulation and make the label mandatory. This study supports the Nutri-Score as an interesting alternative for European countries wishing to implement complementary nutrition labelling on the front of food packaging, and ultimately avoid confusion among consumers through the coexistence of several systems on the European market. 

## Figures and Tables

**Table 1:** Overall distribution (n, %) of the main food groups and subgroups in the Nutri-Score classes, EUROFIR database, 2020.

	Nutri-Score Classes										
	A (Min–1)		B (0–2)		C (3–10)		D (11–18)		E (19–Max)		All
**Egg or egg product**	39	(52,00)	11	(14,67)	10	(13,33)	12	(16,00)	3	(4,00)	75
**Fat or oil**	.	.	.	.	59	(17,56)	214	(63,69)	63	(18,75)	336
Butter or other animal fat	.	.	.	.	1	(1,56)	44	(68,75)	19	(29,69)	64
Margarine or lipid of mixed origin	.	.	.	.	30	(18,87)	95	(59,75)	34	(21,38)	159
Vegetable fat or oil	.	.	.	.	28	(24,78)	75	(66,37)	10	(8,85)	113
**Fruit or fruit product**	420	(73,30)	102	(17,80)	51	(8,90)	.	.	.	.	573
Processed fruit product	80	(35,09)	101	(44,30)	47	(20,61)	.	.	.	.	228
Fresh or unprocessed fruit	340	(98,55)	1	(0,29)	4	(1,16)	.	.	.	.	345
**Grain or grain product**	598	(31,51)	315	(16,60)	329	(17,33)	453	(23,87)	203	(10,70)	1898
Bread and similar products	190	(45,24)	115	(27,38)	91	(21,67)	22	(5,24)	2	(0,48)	420
Breakfast cereal and cereal bar	50	(17,30)	71	(24,57)	69	(23,88)	84	(29,07)	15	(5,19)	289
Cereal or cereal-like milling products and derivatives	130	(72,63)	12	(6,70)	30	(16,76)	5	(2,79)	2	(1,12)	179
Fine bakery ware	4	(0,60)	35	(5,22)	111	(16,54)	337	(50,22)	184	(27,42)	671
Pasta, rice and other cereals	224	(66,08)	82	(24,19)	28	(8,26)	5	(1,47)	.	.	339
**Meat or meat product**	550	(34,81)	236	(14,94)	193	(12,22)	380	(24,05)	221	(13,99)	1587
Meat analogue	5	(45,45)	2	(18,18)	1	(9,09)	2	(18,18)	1	(9,09)	11
Red meat	258	(44,79)	117	(20,31)	91	(15,80)	91	(15,80)	19	(3,30)	576
Poultry meat	137	(46,13)	66	(22,22)	40	(13,47)	49	(16,50)	5	(1,68)	297
Offal and processed meat	154	(21,91)	54	(7,68)	62	(8,82)	237	(33,71)	196	(27,88)	703
**Milk, milk product or milk substitute**	149	(16,45)	201	(22,19)	153	(16,89)	331	(36,53)	72	(7,95)	906
Cheese	24	(6,27)	29	(7,57)	54	(14,10)	234	(61,10)	42	(10,97)	383
Fermented milk product	64	(36,78)	68	(39,08)	39	(22,41)	2	(1,15)	1	(0,57)	174
Frozen dairy dessert	4	(4,71)	3	(3,53)	26	(30,59)	47	(55,29)	5	(5,88)	85
Imitation milk products	19	(35,85)	18	(33,96)	7	(13,21)	6	(11,32)	3	(5,66)	53
Milk	38	(18,01)	83	(39,34)	27	(12,80)	42	(19,91)	21	(9,95)	211
**Composite food product**	526	(19,81)	759	(28,59)	751	(28,29)	502	(18,91)	117	(4,41)	2655
Meat, seafood and egg dish	106	(13,07)	239	(29,47)	225	(27,74)	200	(24,66)	44	(5,43)	811
Potato, pulse, vegetable and savory cereal dish	268	(29,35)	272	(29,79)	239	(26,18)	120	(13,14)	14	(1,53)	913
Prepared salad	83	(60,14)	35	(25,36)	19	(13,77)	1	(0,72)	.	.	138
Sandwich	2	(5,13)	4	(10,26)	15	(38,46)	15	(38,46)	3	(7,69)	39
Savory snack	3	(3,53)	2	(2,35)	13	(15,29)	48	(56,47)	19	(22,35)	85
Soup	49	(14,89)	164	(49,85)	109	(33,13)	5	(1,52)	2	(0,61)	329
Savory sauce, condiment or other ingredient	15	(4,41)	43	(12,65)	134	(39,41)	113	(33,24)	35	(10,29)	340
**Nut, seed or kernel**	72	(40,68)	29	(16,38)	54	(30,51)	21	(11,86)	1	(0,56)	177
Nut or seed product	7	(13,21)	2	(3,77)	23	(43,40)	20	(37,74)	1	(1,89)	53
Unprocessed nut, seed or kernel	65	(52,42)	27	(21,77)	31	(25,00)	1	(0,81)	.	.	124
**Seafood or related product**	427	(50,18)	174	(20,45)	93	(10,93)	143	(16,80)	14	(1,65)	851
Fish or related organism	395	(69,30)	109	(19,12)	34	(5,96)	32	(5,61)	.	.	570
Seafood product	32	(11,39)	65	(23,13)	59	(21,00)	111	(39,50)	14	(4,98)	281
**Sugar or sugar product**	21	(3,42)	54	(8,79)	171	(27,85)	241	(39,25)	127	(20,68)	614
Chocolate or chocolate product	.	.	.	.	5	(3,47)	33	(22,92)	106	(73,61)	144
Jam or marmalade, non-chocolate confectionery or other sugar products	1	(0,52)	5	(2,59)	57	(29,53)	119	(61,66)	11	(5,70)	193
Sugar, honey or syrup	.	.	2	(4,00)	6	(12,00)	42	(84,00)	.	.	50
Dessert and dessert sauce	20	(8,81)	47	(20,70)	103	(45,37)	47	(20,70)	10	(4,41)	227
**Vegetable or vegetable product**	976	(92,95)	39	(3,71)	34	(3,24)	1	(0,10)	.	.	1050
Pulse or pulse product	135	(99,26)	1	(0,74)	.	.	.	.	.	.	136
Starchy root or potato	65	(73,03)	16	(17,98)	8	(8,99)	.	.	.	.	89
Vegetable (excluding potato)	776	(94,06)	22	(2,67)	26	(3,15)	1	(0,12)	.	.	825
**Beverage non-milk**	149	(23,58)	100	(15,82)	108	(17,09)	65	(10,28)	210	(33,23)	632
Juice or nectar	.	.	57	(27,14)	81	(38,57)	25	(11,90)	47	(22,38)	210
Coffee, tea, cocoa	.	.	31	(32,98)	8	(8,51)	9	(9,57)	46	(48,94)	94
Soft drink	.	.	12	(6,70)	19	(10,61)	31	(17,32)	117	(65,36)	179
Water	149	(100,00)	.	.	.	.	.	.	.	.	149

**Table 2:** Discriminating performance of the Nutri-Score in the eight countries.

	Finland		France		Norway		Poland		Portugal		Slovakia		Sweden		Switzerland	
	N ^a^	MC ^b^	N ^a^	MC ^b^	N ^a^	MC ^b^	N ^a^	MC ^b^	N ^a^	MC ^b^	N ^a^	MC ^b^	N ^a^	MC ^b^	N ^a^	MC ^b^
**Egg or egg product**	4	A (62.5)	4	AB (33.33)	3	A (50)	3	A (50)	4	A (45.45)	4	A (66.67)	4	A (55.56)	2	A (66.67)
**Fat or oil**	3	D (54.69)	3	D (69.23)	3	D (63.89)	3	D (62.5)	3	D (62.96)	3	D (71.43)	3	D (66.15)	3	D (59.26)
Butter or other animal fat	2	E (66.67)	2	D (71.43)	2	DE (50)	2	D (80)	2	D (66.67)	1	D (100)	3	DE (42.86)	2	D (83.33)
Margarine or lipid of mixed origin	3	D (60.47)	3	D (65)	2	D (61.11)	2	D (62.5)	3	D (43.75)	2	CE (50)	3	D (71.43)	2	C (70)
Vegetable fat or oil	3	D (50)	3	D (70.83)	3	D (68.75)	2	D (54.55)	1	D (100)	3	D (60)	3	D (62.5)	3	D (72.73)
**Fruit or fruit product**	3	A (88)	3	A (81.08)	3	A (75)	3	A (74.07)	3	A (71.43)	3	A (61.36)	3	A (67.74)	3	A (70.69)
Processed fruit product	3	A (57.14)	3	B (47.62)	3	B (68.18)	3	AB (43.48)	3	C (70)	3	A (42.11)	3	B (48.78)	3	B (52.17)
Fresh or unprocessed fruit	1	A (100)	2	A (98.11)	1	A (100)	2	A (96.77)	1	A (100)	2	A (96.77)	1	A (100)	2	A (94.29)
**Grain or grain product**	5	B (33.74)	5	D (36.83)	5	A (41.91)	5	A (32.72)	5	A (34.04)	5	D (29.58)	5	A (37.29)	5	A (29.38)
Bread and similar products	4	A (49.43)	4	C (49.09)	4	A (71.79)	4	B (53.7)	2	A (70.59)	4	C (61.9)	5	A (49.38)	3	B (48.15)
Breakfast cereal and cereal bar	4	B (67,78)	5	D (46,3)	4	D (50)	4	D (36,36)	4	C (45,45)	4	D (62,07)	4	D (33,33)	5	D (30,23)
Cereal or cereal-like milling products and derivatives	3	A (81.58)	2	A (92.31)	3	A (70)	2	A (87.5)	2	A (70)	4	A (48.28)	5	A (57.69)	2	A (78.57)
Fine bakery ware	5	D (31.2)	4	D (56.55)	5	D (52.22)	3	D (55.56)	4	D (68.57)	4	D (54.55)	4	D (49.35)	4	D (51.25)
Pasta, rice and other cereals	3	A (56,52)	4	A (78)	3	A (68,57)	2	A (94,44)	3	A (47,62)	4	A (63,33)	4	A (55,81)	2	A (93,33)
**Meat or meat product**	5	D (42.18)	5	A (36.04)	5	A (30.07)	5	D (33.33)	5	D (23.98)	5	A (56.32)	5	A (33.89)	5	A (46.46)
Meat analogue	2	AC (50)	1	B (100)	2	AB (50)	.	.	2	DE (50)	.	.	2	A (75)	.	.
Red meat	5	A (43.24)	5	A (49.59)	5	A (35.14)	4	A (38.1)	5	C (28.16)	4	A (64.44)	5	A (49.55)	5	A (65.63)
Poultry meat	5	A (36.11)	5	A (51.67)	5	A (44.44)	4	A (68.42)	4	B (35.48)	4	A (60.71)	5	A (47.62)	3	A (78.57)
Offal and processed meat	5	D (68,06)	5	E (44,37)	5	D (46,34)	5	D (37,18)	5	D (33,33)	5	A (52,14)	5	D (35,46)	5	E (59,18)
**Milk, milk product or milk substitute**	5	B (31.82)	5	D (54.27)	5	D (44.12)	5	B (28.79)	5	B (32.88)	5	A (37.93)	5	D (37.32)	5	D (43.75)
Cheese	5	D (59.52)	5	D (80.53)	4	D (62.75)	5	D (55.17)	5	D (47.83)	5	C (42.86)	5	D (53.7)	5	D (58)
Fermented milk product	3	A (41.86)	3	B (40.54)	4	B (61.11)	2	AB (50)	3	B (60)	3	A (53.85)	5	A (40.74)	3	B (46.15)
Frozen dairy dessert	4	C (46.15)	3	D (77.78)	3	D (50)	1	C (100)	3	CDE (33.33)	.	.	3	D (55)	3	D (61.9)
Imitation milk products	4	B (52.94)	2	B (57.14)	4	DE (33.33)	.	.	1	A (100)	1	A (100)	5	BC (30.77)	2	AB (50)
Milk	5	B (48.72)	5	AB (33.33)	4	D (31.58)	5	B (33.33)	5	B (50)	4	B (50)	5	D (32.14)	5	B (42.31)
**Composite food product**	5	B (31.29)	5	C (31.54)	5	D (31.86)	5	A (44.74)	5	B (30.25)	5	C (38.08)	5	C (29.24)	5	C (47.47)
Meat, seafood and egg dish	5	B (32,22)	5	B (33,77)	5	BC (30,77)	5	D (29,73)	5	C (39,68)	5	C (28,95)	5	B (32,37)	4	B (40)
Potato, pulse, vegetable and savory cereal dish	5	A (38,1)	5	BC (27,5)	4	C (44,12)	4	A (56,72)	5	B (30)	5	C (38,69)	4	B (32,53)	4	BC (45,83)
Prepared salad	3	A (61.84)	3	B (53.85)	1	B (100)	3	A (86.67)	.	.	1	C (100)	4	A (46.15)	2	B (66.67)
Sandwich	2	C (75)	5	D (40.63)	.	.	.	.	.	.	.	.	.	.	1	A (100)
Savory snack	4	D (66.67)	4	D (46.15)	2	D (80)	3	E (50)	3	D (60)	3	D (66.67)	4	D (58.33)	2	D (54.55)
Soup	4	B (44.32)	5	C (44.9)	2	B (60)	2	B (60.47)	3	B (86.36)	3	B (60)	3	B (46)	2	C (74.07)
Savory sauce, condiment or other ingredient	5	C (36,05)	5	C (45,07)	3	D (58,33)	5	D (37,5)	4	E (42,11)	3	C (80)	5	C (44,94)	5	C (35,71)
**Nut, seed or kernel**	4	A (57.14)	4	A (43.9)	4	A (50)	3	A (57.14)	4	A (50)	4	C (40.63)	3	C (60)	5	A (31.58)
Nut or seed product	2	D (75)	3	C (55.56)	2	D (66.67)	1	D (100)	3	A (50)	3	D (50)	3	C (68.42)	4	D (42.86)
Unprocessed nut, seed or kernel	2	A (80)	3	A (53.13)	3	A (60)	2	A (72.73)	3	A (50)	4	C (42.86)	2	A (66.67)	3	A (50)
**Seafood or related product**	4	A (43.94)	5	A (68.28)	5	B (27.81)	4	A (50)	4	A (47.62)	4	A (62.03)	5	A (43.22)	5	A (65.63)
Fish or related organism	3	A (83.33)	4	A (83.62)	4	A (44.09)	3	A (85)	4	A (47.5)	3	A (84)	4	A (77.97)	2	A (90.48)
Seafood product	4	D (52.78)	5	C (30)	5	D (39.47)	3	D (50)	3	A (50)	4	B (34.48)	5	D (54.24)	5	BD (27.27)
**Sugar or sugar product**	5	C (27.12)	5	D (33.33)	4	D (42.59)	4	C (36)	4	D (46.34)	4	D (79.17)	5	D (35)	4	E (41.03)
Chocolate or chocolate product	3	E (66.67)	3	E (83.87)	2	E (86.67)	1	E (100)	3	E (71.43)	2	D (88.24)	3	E (78.57)	3	E (83.33)
Jam or marmalade, non-chocolate confectionery or other sugar products	4	C (50)	4	D (72,73)	4	D (65)	3	C (51,85)	3	D (64,71)	2	D (89,74)	5	D (54,05)	3	D (55,56)
Sugar, honey or syrup	3	D (60)	3	D (75)	1	D (100)	1	D (100)	1	D (100)	1	D (100)	2	D (85.71)	1	D (100)
Dessert and dessert sauce	5	B (35,21)	5	C (56,36)	4	C (57,14)	3	C (57,14)	4	C (57,14)	4	C (54,55)	5	C (47,92)	2	C (85,71)
**Vegetable or vegetable product**	3	A (87.76)	3	A (95.31)	3	A (95.1)	3	A (96.15)	3	A (92.47)	3	A (88.62)	4	A (90.87)	2	A (98.48)
Pulse or pulse product	1	A (100)	1	A (100)	1	A (100)	1	A (100)	1	A (100)	1	A (100)	2	A (96.97)	1	A (100)
Starchy root or potato	3	A (80)	3	A (70.83)	3	A (75)	1	A (100)	3	A (50)	2	B (80)	3	A (88)	2	A (75)
Vegetable (excluding potato)	3	A (87.95)	3	A (97.58)	3	A (96.25)	3	A (95.29)	2	A (96.97)	3	A (90.29)	4	A (90)	1	A (100)
**Beverage non-milk**	5	B (29.17)	5	A (45.45)	3	E (71.43)	4	E (42.65)	5	E (38.46)	5	E (41.33)	5	E (45.83)	5	A (36.99)
Juice or nectar	4	B (36.59)	4	C (29.55)	3	C (50)	4	C (46.94)	3	E (52.94)	4	C (68.42)	4	B (55)	4	C (57.14)
Coffee, tea, cocoa	4	E (47.06)	4	B (50)	2	E (72.73)	2	BE (50)	4	B (46.15)	2	E (75)	4	E (46.67)	3	E (50)
Soft drink	4	E (50)	4	E (45.45)	3	E (88.89)	2	E (93.33)	3	E (50)	3	E (80)	4	E (71.88)	4	E (50)
Water	1	A (100)	1	A (100)	.	.	.	.	1	A (100)	1	A (100)	1	A (100)	1	A (100)

^a^ N is the number of Nutri-Score classes represented in the main food group or subgroup. ^b^ MC (Modal Class) is the predominant Nutri-Score class in the main food group or subgroup. When two Nutri-Score classes were predominant, the two letters are indicated with the corresponding percentage of each.

## Supplementary Materials

The following are available online at https://www.mdpi.com/2072-6643/12/5/1303/s1. Supplemental Material 1: Supplemental methods: EUROFIR food classification. Supplemental Material 2: Flowchart of the food databases used in the present study. Supplemental Material 3: Supplemental Table S1. Distribution of the main food groups and subgroups in the Nutri-Score classes in Finland (*N* = 2075 food products); Supplemental Table S2. Distribution of the main food groups and subgroups in the Nutri-Score classes in France (*N* = 2309 food products); Supplemental Table S3. Distribution of the main food groups and subgroups in the Nutri-Score classes in Norway (*N* = 1101 food products); Supplemental Table S4. Distribution of the main food groups and subgroups in the Nutri-Score classes in Poland (*N* = 926 food products); Supplemental Table S5. Distribution of the main food groups and subgroups in the Nutri-Score classes in Portugal (*N* = 921 food products); Supplemental Table S6. Distribution of the main food groups and subgroups in the Nutri-Score classes in Slovakia (*N* = 1183 food products); Supplemental Table S7. Distribution of the main food groups and subgroups in the Nutri-Score classes in Sweden (*N* = 1990 food products); and Supplemental Table S8. Distribution of the main food groups and subgroups in the Nutri-Score classes in Switzerland (*N* = 849 food products).
