# Supplementary material for: Performance of the Front-of-Pack Nutrition Label Nutri-Score to Discriminate the Nutritional Quality of Foods Products: A Comparative Study across 8 European Countries
Source: Nutrients. 2020 May 2;12(5):1303. doi: 10.3390/nu12051303 (PMC7284849; doi:10.3390/nu12051303)
Supplement: Supplementary file 1 [file nutrients-12-01303-s001.zip › Supplemental Material 1.docx]

**Supplemental methods: EUROFIR food classification**

| **Food group** | **Detailed description** |
| --- | --- |
| **Egg or egg product** | The group includes bird eggs, food product whose predominant constituent is eggs, recipe dishes whose main ingredient is considered to be eggs.  It does not include fish roe (under “Fish”). e.g. chicken eggs, duck eggs, egg yolk. |
| **Fat or oil** | Food substance or component consisting predominantly of mixed glycerol esters of fatty acids and, in far lesser amounts, of fatty acids, sterols and pigments. A fat is solid at room temperature; an oil is liquid at room temperature (20 degrees C).  Excludes essential oils. |
| Butter or other animal fat | (Butter) e.g. butter, butter oil, ghee / (Animal fats) e.g. beef fat, goose fat / (Fish oils) e.g. herring oil, sardine oil. |
| Margarine or lipid of mixed origin | Food product having functional characteristics similar to a butter product; it may be nutritionally equivalent or inferior to the product it purports to resemble. |
| Vegetable fat or oil | In this context, “vegetable fats” are oils that are solid at room temperature (e.g. palm oil, cocoa butter). It may also apply to hydrogenated (hardened) vegetable fats. |
| **Fruit or fruit product** | The group includes: fruits when consumed as a dessert; food products whose predominant constituent is fruit; recipe dishes whose main ingredient is considered to be fruit. A fruit is usually consumed as a dessert when the starch content has been reduced by conversion during ripening to sugars.  The group excludes: vegetable fruits (under “Vegetable”); nuts, seeds and kernels (under *NUT, SEED OR KERNEL PRODUCT*); oils produced from fruits (under *FAT OR OIL*); chutney and pickles (under “Composite foods”). |
| Processed fruit product | Examples are dried fruits (e.g. dried mixed fruit), compotes, canned, stewed fruit (e.g. apple sauce, fruit cocktail). |
| Fresh or unprocessed fruit | Fresh fruits. |
| **Grain or grain product** | This group includes: grains and their milled products obtained from members of the grass family; dough products obtained from grain, such as pasta and breads; breakfast cereals; savoury and sweet products and dishes in which grain products are considered the predominant constituent; substitute flours and other starch products obtained from non-cereal sources. It excludes sweet corn when eaten as a vegetable. |
| Bread and similar products | Products in the “Bread” categories normally have contents of sugars and fat neither exceeding 5% on a dry weight basis. Includes breadcrumbs, bread stuffing, pita bread, matzo, tortilla, wholemeal wheat bread, soda bread, rye bread. |
| Breakfast cereal and cereal bar | Prepared grain product ready or nearly ready for consumption and marketed primarily for breakfast use. Includes formulated breakfast cereals such as 'corn flakes' or ‘muesli’ and simple breakfast cereals such as instant oatmeal.  Excludes rolled oats, corn grits and similar products, which are indexed under *RICE OR OTHER GRAIN *.  Cereal bars include for example flapjack bar, granola bar, muesli bar. |
| Cereal or cereal-like milling products  and derivatives | Examples: wheat flour, wholemeal, substitute flours and starches, wheat flour, patent, soya flour, rye flour, whole, potato flour, cornflour, carob flour, rice flour, arrowroot, buckwheat flour, tapioca. |
| Fine bakery ware | Products in the *BREAD* category normally have contents of sugars and fat neither exceeding 5% on a dry weight basis. Bakery products exceeding either of these limits are termed “Fine bakery ware”. Savoury fine bakery wares will tend to be higher in fat and sweet products in sugars. However, some products, for example scones, may be considered savoury or sweet. Therefore, all of these products are categorised as “Fine bakery wares”, rather than using separate categories for savoury and sweet products.  Some examples are: biscuits, cookies, dry pastry (e.g. savoury biscuits, sweet biscuits and cookies); sweet breads (e.g. croissants, currant bun, dough cakes like muffins or brioche, scone, doughnut); pastry (e.g. danish pastry, baklava); tart, pie (e.g. custard tart, mince pie); cakes (e.g. fruit cake, cream cake, sponge cake. |
| Pasta, rice and other cereals | Pasta can be either dried or fresh, and as main-dish (pasta asciutta) or miniature pasta (e.g. to add to soups). Although pasta is usually made from durum wheat flour, it can also be made from wholemeal flour or buckwheat flour. Noodles contain egg unless specifically referred to as plain noodles. Asian transparent noodles can made from a wide range of flours, many of them noncereal.  For rice and other grain, examples are whole grain wheat, brown rice, bulgur, parboiled rice, rolled oats, wild rice, pearl barley, millet, rolled oats, corn grits and similar products. |
| **Meat or meat product** | This category includes: carcass meat of mammals and birds; offal of mammals and birds; a food product whose predominant constituent is meat; a recipe dish whose main ingredient is considered to be meat. |
| Meat analogue | e.g. textured vegetable protein. |
| Red meat | The group includes carcass meat of domestic animals (e.g. beef, veal, pork, mutton / lamb, horse, rabbit) and game (e.g. wild pig, boar, venison, whale). |
| Poultry meat | The group includes carcass meat of domestic poultry (e.g. chicken, turkey, duck, goose) and game birds (e.g. pheasant, partridge, sea birds) |
| Offal and processed meat | Offal: e.g. liver, kidney, tongue, heart, trotters, giblets.  Processed meat: e.g. ham, bacon, corned beef.  The group includes pastes, pâtés and terrines; sausage meat; dry, smoked sausages (rohwurst); fresh and lightly cooked sausages (bratwurst); cooked sausages (kochwurst); blood & blood products (e.g. haggis, black pudding); other meat products (e.g. galantine, brawn). |
| **Milk, milk product or milk substitute** | Liquid milks and processed milks; cream; milk products including fermented milk products, yoghurts and cheeses; milk product substitutes (e.g. made from soya); milk beverage powders; dairy ice cream. The category does not include butter and butter spreads (under *FAT OR OIL*); sauces and soups with a milk product as the main ingredient (under *PREPARED FOOD PRODUCT*). |
| Cheese | Cheese is the ripened or unripened soft or semi-hard, hard and extra hard product, which may be coated, and in which the whey protein/casein ratio does not exceed that of milk, obtained by : coagulating wholly or partly … Through the action of rennet or other suitable coagulating agents, and by partially draining the whey resulting from such coagulation; and/or processing techniques involving coagulation of the protein of milk and/or products obtained from milk which give an end-product with similar physical, chemical and organoleptic characteristics (CODEX STAN A-6-1978, Rev.1-1999, Amended 2003).  The group includes goat and sheep cheeses, and cheeses made from sour milk, whey or buttermilk. |
| Fermented milk product | Fermented milk is a milk product obtained by fermentation of milk, which milk may have been manufactured from products obtained from milk with or without compositional modification as limited by the provision in Section 3.3, by the action of suitable microorganisms and resulting in reduction of pH with or without coagulation (CODEX STAN 243-2003). Fermented milk products include a range of foods commonly referred to as yogurt (or yoghurt), plus sour milk drinks produced by fermentation. A few of these contain alcohol as they are made with combined lactic and yeast ferments (e.g. kefir, koumiss); others are lactic fermented milk products (e.g. cieddu, kaeder milk, skyr, taette). In some traditional fermented milk products, such as Stragisto (strained yoghurt), Labneh, Ymer and Ylette, the protein has been increased to minimum 5.6%. |
| Frozen dairy dessert | A frozen dessert prepared from one or more dairy ingredients plus other ingredients. SN: Includes frozen dairy items offered in a cone, a sandwich or as a cake or pie, such as frozen yogurt in a cone or an ice cream sandwich; these are also indexed under *GRAIN ADDED*. Non-dairy ices (e.g. Water ices, granitas, sorbets) are classified under *DESSERT*. |
| Immitation milk products | The group includes soya milk, soya cheese, non-dairy coffee creamer. |
| Milk | Milk in all forms, milk-based beverage, cultured milk product,  or milk. Includes fresh cream, crème fraîche and sour cream. Or liquid milks are the secretion of the mammary gland of animals such as cow, sheep, goat, buffalo and camel, and include Human milk. The category includes milks that have only been processed for reasons of food safety (e.g. pasteurisation), preservation (e.g. UHT) or skimming to reduce fat content. Or Processed milks are milks that have been subject to processing that modifies their consistency (e.g. evaporated milk) and/or composition other than fat content (e.g. whey). The group also includes milk-based drinks like milkshakes. |
| **Composite food product** | Use for foods and ingredients that could not fit into any of the above classes. |
| Meat, seafood and egg dish | Egg dish: e.g. omelette, soufflé, meringue, eggnog.  Meat dish: e.g. meat burger, meat balls, meat pie or pasty.  Seafood dish: recipe dish whose main ingredient is considered to be fish. |
| Potato, pulse, vegetable and savoury cereal dish | Potato dish: a recipe dish whose main ingredient is considered to be potatoes.  Pulse dish: a recipe dish whose main ingredient is considered to be pulses.  Savoury cereal dish includes dumpling, risotto, savoury pancake, pizza, couscous, savoury pie, sandwich.  Vegetable dish: a recipe dish whose main ingredient is considered to be vegetables. |
| Prepared salad | A combination of one or more vegetable, fruit, herb, meat, poultry, seafood, egg, cereal or pasta, usually served with some kind of moist dressing; may be moulded with a jellying agent. Salad takes precedence over other product types. Examples are egg salad, tuna salad, mixed vegetable salad, jelled fruit salad, macaroni salad, potato salad, rice salad, mayonnaise salad. |
| Sandwich | A sandwich is a food item made of two or more slices of leavened bread with one or more layers of filling, typically meat or cheese, with the addition of vegetables or salad. The bread can be used as is, or it can be coated with butter, oil, mustard or other condiments to enhance flavor and texture. Includes cheese-based sandwich filling, fish-based sandwich filling. |
| Savoury snack | Unsweetened food product marketed for consumption between meals; excludes nuts, edible seeds, and sweetened products such as cakes, puddings and candies. Examples are potato crisps, maize-based snacks, pretzels, popcorn. |
| Soup | A liquid food made by simmering meat, poultry, seafood or vegetables, being clear or thickened to the consistency of a thin puree or having milk or cream added, and often containing pieces of solid food such as meat, shellfish, pasta or vegetables. Soup takes precedence over other food products.  Examples are egg and lemon soup, oxtail soup, fish soup, rice soup, lentil soup, minestrone, cherry soup. |
| Savoury sauce, condiment or other ingredient | Savoury sauce: nonstandardized food product used as a meal accompaniment and consisting of a mixture of fats or oils, starch, liquid and other optional ingredients specified by the recipe; excludes condiments. Includes. bolognese sauce , white sauce, brown sauce, butter sauce, tomato sauce. Sauce is a very general term for a liquid or semi liquid seasoning or other accompaniment for food.  When sauces are cooked as part of, or adjuncts to, dishes (including starters, main courses and desserts), they have been assigned to the *SAVOURY SAUCE* or *DESSERT SAUCE* subgroups, which include ready-prepared retail products as well as sauces prepared in the kitchen (above, at, or below room temperature).  Condiment of other ingredient: this subgroup includes baking goods and other ingredients such as flavourings, essence and extracts which are difficult to assign to other main groups; dressings, condiments and mixed accompaniments such as chutneys and pickles. The group does not include starches (under *GRAIN OR GRAIN PRODUCT*). |
| **Nut, seed or kernel** | The group includes nuts, seeds and kernels in all forms, including pastes. |
| Nut or seed product | e.g. coconut milk, chestnut purée, tahini paste, peanut butter. |
| Nut, seed or kernel unprocessed | e.g. walnut, hazelnut, sweet chestnut, sunflower seed, olive seed, pine nut, apricot kernel, peanuts. |
| **Seafood or related product** | The group includes marine or freshwater fish, molluscs, crustaceans, and other fauna such as reptiles, insects or frogs not in the “Meats” group. The group also includes seafood product analogs and seafood-based sausage or luncheon meat as well as such products as squid ink and clam juice. |
| Fish or related organism | Flesh from marine or freshwater fish, molluscs, crustaceans, and other fauna such as reptiles, insects or frogs not in the “Meats” group. |
| Seafood product | Includes fish offal; a food product whose predominant constituent is fish (e.g. dried and salted fish, smoked fish, canned fish, pickled fish, restructured fish and fish analogues, surimi; fish paste, pâté). |
| **Sugar or sugar product** | This group includes sucrose and other sugars, sugar substitutes, honey and syrups; fruit jams, marmalades and other spreads; dessert jellies and toppings; chocolate and non-chocolate confectionery; a food product whose predominant constituent is sugar or chocolate; a recipe dish whose main ingredient is considered to be sugar orchocolate. |
| Chocolate or chocolate product | Some examples are chocolate slab or bar (e.g. milk chocolate bar, white chocolate bar), filled chocolate candy, chocolatecoated confectionery bars. |
| Jam or marmelade, non-chocolate confectionery or other  sugar product | Semisolid or jelled food prepared from fruit or fruit juice and other ingredients. The group includes fruit jam, fruit jelly preserve, marmalade.  Some examples of non-chocolate confectionery or other sugar products are boiled sweet, gum sweet, nougat, turkish delight, chewing gum, marzipan, candied fruit. |
| Sugar, honey or syrup | This group includes sugars (e.g. white sugar, brown sugar, fructose), sugar substitutes (non-nutritive sweeteners like aspartame & saccharine, nutritive sweeteners like sorbitol & mannitol), honey, syrups (e.g. molasses, maple syrup, corn syrup). |
| Dessert and dessert sauce | Sweetened prepared product usually consumed after the main course in a meal. Excludes fruit or fruit products, bakery products and confectionery. Includes sweet puddings (custards, starch puddings), nondairy ices (e.g. water ices, granitas, sorbets) and gelatine desserts.  Sweetened and flavored product that is used as an accompaniment to desserts, e.g. fruit sauce, fudge sauce, brandy sauce. Sauce is a very general term for a liquid or semi liquid seasoning or other accompaniment for food. When sauces are cooked as part of, or adjuncts to, dishes (including starters, main courses and desserts), they have been assigned to the *SAVOURY SAUCE* or *DESSERT SAUCE* subgroups, which include ready-prepared retail products as well as sauces prepared in the kitchen (above, at, or below room temperature). |
| **Vegetable or vegetable product** | Plants and parts of plants eaten as vegetables (i.e. normally consumed as a savoury and usually with other foods as sources of protein and/or grain starch), including immature pulses; edible fungi and seaweed; a food product whose predominant constituent is vegetables; a recipe dish whose main ingredient is considered to be vegetables.  The group excludes : fruiting body of a plant when this is consumed as a dessert fruit (under *FRUIT*); seeds, kernels and nuts (under *NUT, SEED OR KERNEL PRODUCT*); oils produced from vegetable plants (under *FAT OR OIL*); herbs, spices, chutney and pickles produced from vegetables (under *SPICE, CONDIMENT OR OTHER INGREDIENT*); food products produced wholly or partially from vegetables but used as a substitute for a food assigned to another main group (for example, potato flour is a substitute flour under *FLOUR OR STARCH*). |
| Pulse or pulse product | Use for crops harvested as dry seed. Index green beans and green peas as vegetables. |
| Starchy root or potato | Potatoes were excluded distinguished from other vegetables because of their high starch content. Some examples of foods classified here would be new potato, main-crop potato, Jerusalem artichoke, sweet potato, yam. |
| Vegetable (excluding potato) | Potatoes were excluded distinguished from other vegetables because of their high starch content. Herbs have been included where these may be consumed in significant amounts as vegetables, either raw or cooked (e.g. parsley, chives). A vegetable fruit is usually consumed as a vegetable when the starch content is high (e.g. avocado, olive).  Some examples of foods classified here would be lettuce, cabbage, rhubarb, asparagus, onion, carrot. |
| **Beverage non-milk** | Alcoholic or non-alcoholic beverage; excludes milk and milkbased beverages. |
| Juice or nectar | The members of the work group in Action COST 99/Eurofoods considered it important to be able to separate fruit juices from both *NON-ALCOHOLIC BEVERAGE* and *FRUIT* in the EFG classification, in order to evaluate and compare consumption patterns across countries.  Examples are orange juice, apple juice, tomato juice, fruit and vegetable nectars. |
| Coffee, tea, cocoa | Beverage prepared by extracting flavour and other components from food sources by percolation and/or immersion in water, usually at near-boiling temperature.  The group includes coffee (e.g. instant coffee, coffee and chicory essence), tea, herbal tea (e.g. green tea, black tea, tisane), cocoa beverage and beverage powder. |
| Soft drink | Including carbonated soft drinks (e.g. soda water, carbonated lemonade, cola, tonic), non-dilution still drinks (e.g. still lemonade), dilution drinks (concentrates which are diluted with water prior to consumption , e.g. rosehip syrup, fruit squash, lime cordial). |
| Water | Including tap water, carbonated mineral water, still mineral water. |
