# Supplementary material for: Performance of the Front-of-Pack Nutrition Label Nutri-Score to Discriminate the Nutritional Quality of Foods Products: A Comparative Study across 8 European Countries
Source: Nutrients. 2020 May 2;12(5):1303. doi: 10.3390/nu12051303 (PMC7284849; doi:10.3390/nu12051303)
Supplement: Supplementary file 1 [file nutrients-12-01303-s001.zip › Supplemental Material 2 -revised.docx]

Finland

N=2153

Norway

N=1188

Poland

N=932

Portugal

N=962

Slovakia

N=1263

Sweden

N=2056

Switzerland

N=928

France

N=2805

Suppression of products with invalid or missing nutritional values

N=478 foods

Finland

N=2149

France

N=2438

Norway

N=1127

Poland

N=932

Portugal

N=962

Slovakia

N=1263

Sweden

N=2056

Switzerland

N=882

N=12287 foods

N=11809 foods

Suppression of food groups excluded from the labelling regulation

N=462 foods

Finland

N=2075

France

N=2309

Norway

N=1101

Poland

N=919

Portugal

N=921

Slovakia

N=1183

Sweden

N=1990

Switzerland

N=849

N=11347 foods

**Supplemental Figure 1. Flowchart of the food databases used in the present study**
