# Supplementary material for: Performance of the Front-of-Pack Nutrition Label Nutri-Score to Discriminate the Nutritional Quality of Foods Products: A Comparative Study across 8 European Countries
Source: Nutrients. 2020 May 2;12(5):1303. doi: 10.3390/nu12051303 (PMC7284849; doi:10.3390/nu12051303)
Supplement: Supplementary file 1 [file nutrients-12-01303-s001.zip › Supplemental Material 3 - revised.docx]

**Supplemental Table 1. Distribution of the main food groups and subgroups in the Nutri-Score classes in Finland (N=2075 food products)**

|  | **Nutri-Score classes** | | | | | | | | | | |
| --- | --- | --- | --- | --- | --- | --- | --- | --- | --- | --- | --- |
|  | **A [Min - -1]** | | **B [0 - 2]** | | **C [3 - 10]** | | **D [11 - 18]** | | **E [19 - Max]** | | **All** |
| **Egg or egg product** | **5** | (62,50) | **1** | (12,50) | **1** | (12,50) | **1** | (12,50) | **.** | . | **8** |
| **Fat or oil** | **.** | . | **.** | . | **10** | (15,63) | **35** | (54,69) | **19** | (29,69) | **64** |
| Butter or other animal fat | **.** | . | **.** | . | **.** | . | **3** | (33,33) | **6** | (66,67) | **9** |
| Margarine or lipid of mixed origin | **.** | . | **.** | . | **6** | (13,95) | **26** | (60,47) | **11** | (25,58) | **43** |
| Vegetable fat or oil | **.** | . | **.** | . | **4** | (33,33) | **6** | (50,00) | **2** | (16,67) | **12** |
| **Fruit or fruit product** | **66** | (88,00) | **7** | (9,33) | **2** | (2,67) | **.** | . | **.** | . | **75** |
| Processed fruit product | **12** | (57,14) | **7** | (33,33) | **2** | (9,52) | **.** | . | **.** | . | **21** |
| Fresh or unprocessed fruit | **54** | (100,00) | **.** | . | **.** | . | **.** | . | **.** | . | **54** |
| **Grain or grain product** | **127** | (31,05) | **138** | (33,74) | **70** | (17,11) | **45** | (11,00) | **29** | (7,09) | **409** |
| Bread and similar products | **43** | (49,43) | **31** | (35,63) | **9** | (10,34) | **4** | (4,60) | **.** | . | **87** |
| Breakfast cereal and cereal bar | **13** | (14,44) | **61** | (67,78) | **14** | (15,56) | **2** | (2,22) | **.** | . | **90** |
| Cereal or cereal-like milling products and derivatives | **31** | (81,58) | **2** | (5,26) | **5** | (13,16) | **.** | . | **.** | . | **38** |
| Fine bakery ware | **1** | (0,80) | **18** | (14,40) | **38** | (30,40) | **39** | (31,20) | **29** | (23,20) | **125** |
| Pasta, rice and other cereals | **39** | (56,52) | **26** | (37,68) | **4** | (5,80) | **.** | . | **.** | . | **69** |
| **Meat or meat product** | **38** | (25,85) | **17** | (11,56) | **20** | (13,61) | **62** | (42,18) | **10** | (6,80) | **147** |
| Meat analogue | **1** | (50,00) | **.** | . | **1** | (50,00) | **.** | . | **.** | . | **2** |
| Red meat | **16** | (43,24) | **10** | (27,03) | **7** | (18,92) | **3** | (8,11) | **1** | (2,70) | **37** |
| Poultry meat | **13** | (36,11) | **5** | (13,89) | **7** | (19,44) | **10** | (27,78) | **1** | (2,78) | **36** |
| Offal and processed meat | **8** | (11,11) | **2** | (2,78) | **5** | (6,94) | **49** | (68,06) | **8** | (11,11) | **72** |
| **Milk, milk product or milk substitute** | **27** | (17,53) | **49** | (31,82) | **33** | (21,43) | **38** | (24,68) | **7** | (4,55) | **154** |
| Cheese | **1** | (2,38) | **4** | (9,52) | **9** | (21,43) | **25** | (59,52) | **3** | (7,14) | **42** |
| Fermented milk product | **18** | (41,86) | **15** | (34,88) | **10** | (23,26) | **.** | . | **.** | . | **43** |
| Frozen dairy dessert | **.** | . | **2** | (15,38) | **6** | (46,15) | **4** | (30,77) | **1** | (7,69) | **13** |
| Immitation milk products | **5** | (29,41) | **9** | (52,94) | **2** | (11,76) | **1** | (5,88) | **.** | . | **17** |
| Milk | **3** | (7,69) | **19** | (48,72) | **6** | (15,38) | **8** | (20,51) | **3** | (7,69) | **39** |
| **Composite food product** | **227** | (26,71) | **266** | (31,29) | **212** | (24,94) | **125** | (14,71) | **20** | (2,35) | **850** |
| Meat, seafood and egg dish | **30** | (11,11) | **87** | (32,22) | **75** | (27,78) | **63** | (23,33) | **15** | (5,56) | **270** |
| Potato, pulse, vegetable and savoury cereal dish | **120** | (38,10) | **101** | (32,06) | **66** | (20,95) | **27** | (8,57) | **1** | (0,32) | **315** |
| Prepared salad | **47** | (61,84) | **19** | (25,00) | **10** | (13,16) | **.** | . | **.** | . | **76** |
| Sandwich | **.** | . | **1** | (16,67) | **3** | (50,00) | **2** | (33,33) | **.** | . | **6** |
| Savoury snack | **.** | . | **1** | (11,11) | **1** | (11,11) | **6** | (66,67) | **1** | (11,11) | **9** |
| Soup | **21** | (23,86) | **39** | (44,32) | **26** | (29,55) | **2** | (2,27) | **.** | . | **88** |
| Savoury sauce, condiment or other ingredient | **9** | (10,47) | **18** | (20,93) | **31** | (36,05) | **25** | (29,07) | **3** | (3,49) | **86** |
| **Nut, seed or kernel** | **8** | (57,14) | **2** | (14,29) | **1** | (7,14) | **3** | (21,43) | **.** | . | **14** |
| Nut or seed product | **.** | . | **.** | . | **1** | (25,00) | **3** | (75,00) | **.** | . | **4** |
| Unprocessed nut, seed or kernel | **8** | (80,00) | **2** | (20,00) | **.** | . | **.** | . | **.** | . | **10** |
| **Seafood or related product** | **29** | (43,94) | **7** | (10,61) | **9** | (13,64) | **21** | (31,82) | **.** | . | **66** |
| Fish or related organism | **25** | (83,33) | **3** | (10,00) | **.** | . | **2** | (6,67) | **.** | . | **30** |
| Seafood product | **4** | (11,11) | **4** | (11,11) | **9** | (25,00) | **19** | (52,78) | **.** | . | **36** |
| **Sugar or sugar product** | **12** | (10,17) | **28** | (23,73) | **32** | (27,12) | **31** | (26,27) | **15** | (12,71) | **118** |
| Chocolate or chocolate product | **.** | . | **.** | . | **1** | (6,67) | **4** | (26,67) | **10** | (66,67) | **15** |
| Jam or marmalade, non-chocolate confectionery or other sugar products | **.** | . | **2** | (9,09) | **11** | (50,00) | **7** | (31,82) | **2** | (9,09) | **22** |
| Sugar, honey or syrup | **.** | . | **1** | (10,00) | **3** | (30,00) | **6** | (60,00) | **.** | . | **10** |
| Dessert and dessert sauce | **12** | (16,90) | **25** | (35,21) | **17** | (23,94) | **14** | (19,72) | **3** | (4,23) | **71** |
| **Vegetable or vegetable product** | **86** | (87,76) | **5** | (5,10) | **7** | (7,14) | **.** | . | **.** | . | **98** |
| Pulse or pulse product | **5** | (100,00) | **.** | . | **.** | . | **.** | . | **.** | . | **5** |
| Starchy root or potato | **8** | (80,00) | **1** | (10,00) | **1** | (10,00) | **.** | . | **.** | . | **10** |
| Vegetable (excluding potato) | **73** | (87,95) | **4** | (4,82) | **6** | (7,23) | **.** | . | **.** | . | **83** |
| **Beverage non-milk** | **4** | (5,56) | **21** | (29,17) | **17** | (23,61) | **10** | (13,89) | **20** | (27,78) | **72** |
| Juice or nectar | **.** | . | **15** | (36,59) | **13** | (31,71) | **6** | (14,63) | **7** | (17,07) | **41** |
| Coffee, tea, cocoa | **.** | . | **3** | (17,65) | **3** | (17,65) | **3** | (17,65) | **8** | (47,06) | **17** |
| Soft drink | **.** | . | **3** | (30,00) | **1** | (10,00) | **1** | (10,00) | **5** | (50,00) | **10** |
| Water | **4** | (100,00) | **.** | . | **.** | . | **.** | . | **.** | . | **4** |

**Supplemental Table 2. Distribution of the main food groups and subgroups in the Nutri-Score classes in France (N=2309 food products)**

|  | **Nutri-Score classes** | | | | | | | | | | |
| --- | --- | --- | --- | --- | --- | --- | --- | --- | --- | --- | --- |
|  | **A [Min - -1]** | | **B [0 - 2]** | | **C [3 - 10]** | | **D [11 - 18]** | | **E [19 - Max]** | | **All** |
| **Egg or egg product** | **5** | (33,33) | **5** | (33,33) | **.** | . | **3** | (20,00) | **2** | (13,33) | **15** |
| **Fat or oil** | **.** | . | **.** | . | **12** | (18,46) | **45** | (69,23) | **8** | (12,31) | **65** |
| Butter or other animal fat | **.** | . | **.** | . | **.** | . | **15** | (71,43) | **6** | (28,57) | **21** |
| Margarine or lipid of mixed origin | **.** | . | **.** | . | **6** | (30,00) | **13** | (65,00) | **1** | (5,00) | **20** |
| Vegetable fat or oil | **.** | . | **.** | . | **6** | (25,00) | **17** | (70,83) | **1** | (4,17) | **24** |
| **Fruit or fruit product** | **60** | (81,08) | **11** | (14,86) | **3** | (4,05) | **.** | . | **.** | . | **74** |
| Processed fruit product | **8** | (38,10) | **10** | (47,62) | **3** | (14,29) | **.** | . | **.** | . | **21** |
| Fresh or unprocessed fruit | **52** | (98,11) | **1** | (1,89) | **.** | . | **.** | . | **.** | . | **53** |
| **Grain or grain product** | **79** | (22,38) | **17** | (4,82) | **67** | (18,98) | **130** | (36,83) | **60** | (17,00) | **353** |
| Bread and similar products | **12** | (21,82) | **7** | (12,73) | **27** | (49,09) | **9** | (16,36) | **.** | . | **55** |
| Breakfast cereal | **4** | (7,41) | **2** | (3,70) | **21** | (38,89) | **25** | (46,30) | **2** | (3,70) | **54** |
| Cereal or cereal-like milling products and derivatives | **24** | (92,31) | **.** | . | **2** | (7,69) | **.** | . | **.** | . | **26** |
| Fine bakery ware | **.** | . | **2** | (1,19) | **13** | (7,74) | **95** | (56,55) | **58** | (34,52) | **168** |
| Pasta, rice and other cereals | **39** | (78,00) | **6** | (12,00) | **4** | (8,00) | **1** | (2,00) | **.** | . | **50** |
| **Meat or meat product** | **120** | (36,04) | **50** | (15,02) | **36** | (10,81) | **54** | (16,22) | **73** | (21,92) | **333** |
| Meat analogue | **.** | . | **1** | (100,00) | **.** | . | **.** | . | **.** | . | **1** |
| Red meat | **60** | (49,59) | **28** | (23,14) | **16** | (13,22) | **12** | (9,92) | **5** | (4,13) | **121** |
| Poultry meat | **31** | (51,67) | **15** | (25,00) | **7** | (11,67) | **6** | (10,00) | **1** | (1,67) | **60** |
| Offal and processed meat | **29** | (19,21) | **6** | (3,97) | **13** | (8,61) | **36** | (23,84) | **67** | (44,37) | **151** |
| **Milk, milk product or milk substitute** | **25** | (12,56) | **31** | (15,58) | **24** | (12,06) | **108** | (54,27) | **11** | (5,53) | **199** |
| Cheese | **3** | (2,65) | **4** | (3,54) | **9** | (7,96) | **91** | (80,53) | **6** | (5,31) | **113** |
| Fermented milk product | **11** | (29,73) | **15** | (40,54) | **11** | (29,73) | **.** | . | **.** | . | **37** |
| Frozen dairy dessert | **.** | . | **.** | . | **2** | (11,11) | **14** | (77,78) | **2** | (11,11) | **18** |
| Immitation milk products | **3** | (42,86) | **4** | (57,14) | **.** | . | **.** | . | **.** | . | **7** |
| Milk | **8** | (33,33) | **8** | (33,33) | **2** | (8,33) | **3** | (12,50) | **3** | (12,50) | **24** |
| **Composite food product** | **66** | (15,42) | **109** | (25,47) | **135** | (31,54) | **97** | (22,66) | **21** | (4,91) | **428** |
| Meat, seafood and egg dish | **14** | (18,18) | **26** | (33,77) | **18** | (23,38) | **18** | (23,38) | **1** | (1,30) | **77** |
| Potato, pulse, vegetable and savoury cereal dish | **42** | (26,25) | **44** | (27,50) | **44** | (27,50) | **27** | (16,88) | **3** | (1,88) | **160** |
| Prepared salad | **4** | (30,77) | **7** | (53,85) | **2** | (15,38) | **.** | . | **.** | . | **13** |
| Sandwich | **1** | (3,13) | **3** | (9,38) | **12** | (37,50) | **13** | (40,63) | **3** | (9,38) | **32** |
| Savoury snack | **1** | (3,85) | **.** | . | **5** | (19,23) | **12** | (46,15) | **8** | (30,77) | **26** |
| Soup | **2** | (4,08) | **20** | (40,82) | **22** | (44,90) | **3** | (6,12) | **2** | (4,08) | **49** |
| Savoury sauce, condiment or other ingredient | **2** | (2,82) | **9** | (12,68) | **32** | (45,07) | **24** | (33,80) | **4** | (5,63) | **71** |
| **Nut, seed or kernel** | **18** | (43,90) | **6** | (14,63) | **14** | (34,15) | **3** | (7,32) | **.** | . | **41** |
| Nut or seed product | **1** | (11,11) | **.** | . | **5** | (55,56) | **3** | (33,33) | **.** | . | **9** |
| Unprocessed nut, seed or kernel | **17** | (53,13) | **6** | (18,75) | **9** | (28,13) | **.** | . | **.** | . | **32** |
| **Seafood or related product** | **155** | (68,28) | **37** | (16,30) | **17** | (7,49) | **16** | (7,05) | **2** | (0,88) | **227** |
| Fish or related organism | **148** | (83,62) | **24** | (13,56) | **2** | (1,13) | **3** | (1,69) | **.** | . | **177** |
| Seafood product | **7** | (14,00) | **13** | (26,00) | **15** | (30,00) | **13** | (26,00) | **2** | (4,00) | **50** |
| **Sugar or sugar product** | **5** | (4,17) | **9** | (7,50) | **37** | (30,83) | **40** | (33,33) | **29** | (24,17) | **120** |
| Chocolate or chocolate product | **.** | . | **.** | . | **1** | (3,23) | **4** | (12,90) | **26** | (83,87) | **31** |
| Jam or marmalade, non-chocolate confectionery or other sugar products | **.** | . | **1** | (4,55) | **3** | (13,64) | **16** | (72,73) | **2** | (9,09) | **22** |
| Sugar, honey or syrup | **.** | . | **1** | (8,33) | **2** | (16,67) | **9** | (75,00) | **.** | . | **12** |
| Dessert and dessert sauce | **5** | (9,09) | **7** | (12,73) | **31** | (56,36) | **11** | (20,00) | **1** | (1,82) | **55** |
| **Vegetable or vegetable product** | **244** | (95,31) | **8** | (3,13) | **4** | (1,56) | **.** | . | **.** | . | **256** |
| Pulse or pulse product | **25** | (100,00) | **.** | . | **.** | . | **.** | . | **.** | . | **25** |
| Starchy root or potato | **17** | (70,83) | **5** | (20,83) | **2** | (8,33) | **.** | . | **.** | . | **24** |
| Vegetable (excluding potato) | **202** | (97,58) | **3** | (1,45) | **2** | (0,97) | **.** | . | **.** | . | **207** |
| **Beverage non-milk** | **90** | (45,45) | **22** | (11,11) | **23** | (11,62) | **23** | (11,62) | **40** | (20,20) | **198** |
| Juice or nectar | **.** | . | **10** | (22,73) | **13** | (29,55) | **9** | (20,45) | **12** | (27,27) | **44** |
| Coffee, tea, cocoa | **.** | . | **10** | (50,00) | **1** | (5,00) | **1** | (5,00) | **8** | (40,00) | **20** |
| Soft drink | **.** | . | **2** | (4,55) | **9** | (20,45) | **13** | (29,55) | **20** | (45,45) | **44** |
| Water | **90** | (100,00) | **.** | . | **.** | . | **.** | . | **.** | . | **90** |

**Supplemental Table 3. Distribution of the main food groups and subgroups in the Nutri-Score classes in Norway (N=1101 food products)**

|  | **Nutri-Score classes** | | | | | | | | | | |
| --- | --- | --- | --- | --- | --- | --- | --- | --- | --- | --- | --- |
|  | **A [Min - -1]** | | **B [0 - 2]** | | **C [3 - 10]** | | **D [11 - 18]** | | **E [19 - Max]** | | **All** |
| **Egg or egg product** | **5** | (50,00) | **2** | (20,00) | **3** | (30,00) | **.** | . | **.** | . | **10** |
| **Fat or oil** | **.** | . | **.** | . | **3** | (8,33) | **23** | (63,89) | **10** | (27,78) | **36** |
| Butter or other animal fat | **.** | . | **.** | . | **.** | . | **1** | (50,00) | **1** | (50,00) | **2** |
| Margarine or lipid of mixed origin | **.** | . | **.** | . | **.** | . | **11** | (61,11) | **7** | (38,89) | **18** |
| Vegetable fat or oil | **.** | . | **.** | . | **3** | (18,75) | **11** | (68,75) | **2** | (12,50) | **16** |
| **Fruit or fruit product** | **51** | (75,00) | **15** | (22,06) | **2** | (2,94) | **.** | . | **.** | . | **68** |
| Processed fruit product | **5** | (22,73) | **15** | (68,18) | **2** | (9,09) | **.** | . | **.** | . | **22** |
| Fresh or unprocessed fruit | **46** | (100,00) | **.** | . | **.** | . | **.** | . | **.** | . | **46** |
| **Grain or grain product** | **101** | (41,91) | **28** | (11,62) | **35** | (14,52) | **57** | (23,65) | **20** | (8,30) | **241** |
| Bread and similar products | **56** | (71,79) | **9** | (11,54) | **12** | (15,38) | **1** | (1,28) | **.** | . | **78** |
| Breakfast cereal | **4** | (22,22) | **3** | (16,67) | **2** | (11,11) | **9** | (50,00) | **.** | . | **18** |
| Cereal or cereal-like milling products and derivatives | **14** | (70,00) | **1** | (5,00) | **5** | (25,00) | **.** | . | **.** | . | **20** |
| Fine bakery ware | **3** | (3,33) | **6** | (6,67) | **14** | (15,56) | **47** | (52,22) | **20** | (22,22) | **90** |
| Pasta, rice and other cereals | **24** | (68,57) | **9** | (25,71) | **2** | (5,71) | **.** | . | **.** | . | **35** |
| **Meat or meat product** | **46** | (30,07) | **29** | (18,95) | **10** | (6,54) | **45** | (29,41) | **23** | (15,03) | **153** |
| Meat analogue | **1** | (50,00) | **1** | (50,00) | **.** | . | **.** | . | **.** | . | **2** |
| Red meat | **26** | (35,14) | **18** | (24,32) | **7** | (9,46) | **18** | (24,32) | **5** | (6,76) | **74** |
| Poultry meat | **16** | (44,44) | **9** | (25,00) | **1** | (2,78) | **8** | (22,22) | **2** | (5,56) | **36** |
| Offal and processed meat | **3** | (7,32) | **1** | (2,44) | **2** | (4,88) | **19** | (46,34) | **16** | (39,02) | **41** |
| **Milk, milk product or milk substitute** | **11** | (10,78) | **16** | (15,69) | **15** | (14,71) | **45** | (44,12) | **15** | (14,71) | **102** |
| Cheese | **2** | (3,92) | **.** | . | **5** | (9,80) | **32** | (62,75) | **12** | (23,53) | **51** |
| Fermented milk product | **3** | (16,67) | **11** | (61,11) | **3** | (16,67) | **1** | (5,56) | **.** | . | **18** |
| Frozen dairy dessert | **.** | . | **.** | . | **3** | (37,50) | **4** | (50,00) | **1** | (12,50) | **8** |
| Immitation milk products | **1** | (16,67) | **.** | . | **1** | (16,67) | **2** | (33,33) | **2** | (33,33) | **6** |
| Milk | **5** | (26,32) | **5** | (26,32) | **3** | (15,79) | **6** | (31,58) | **.** | . | **19** |
| **Composite food product** | **8** | (7,08) | **27** | (23,89) | **33** | (29,20) | **36** | (31,86) | **9** | (7,96) | **113** |
| Meat, seafood and egg dish | **4** | (10,26) | **12** | (30,77) | **12** | (30,77) | **10** | (25,64) | **1** | (2,56) | **39** |
| Potato, pulse, vegetable and savoury cereal dish | **4** | (11,76) | **11** | (32,35) | **15** | (44,12) | **4** | (11,76) | **.** | . | **34** |
| Prepared salad | **.** | . | **1** | (100,00) | **.** | . | **.** | . | **.** | . | **1** |
| Savoury snack | **.** | . | **.** | . | **.** | . | **8** | (80,00) | **2** | (20,00) | **10** |
| Soup | **.** | . | **3** | (60,00) | **2** | (40,00) | **.** | . | **.** | . | **5** |
| Savoury sauce, condiment or other ingredient | **.** | . | **.** | . | **4** | (16,67) | **14** | (58,33) | **6** | (25,00) | **24** |
| **Nut, seed or kernel** | **9** | (50,00) | **3** | (16,67) | **4** | (22,22) | **2** | (11,11) | **.** | . | **18** |
| Nut or seed product | **.** | . | **.** | . | **1** | (33,33) | **2** | (66,67) | **.** | . | **3** |
| Unprocessed nut, seed or kernel | **9** | (60,00) | **3** | (20,00) | **3** | (20,00) | **.** | . | **.** | . | **15** |
| **Seafood or related product** | **45** | (26,63) | **47** | (27,81) | **32** | (18,93) | **38** | (22,49) | **7** | (4,14) | **169** |
| Fish or related organism | **41** | (44,09) | **28** | (30,11) | **16** | (17,20) | **8** | (8,60) | **.** | . | **93** |
| Seafood product | **4** | (5,26) | **19** | (25,00) | **16** | (21,05) | **30** | (39,47) | **7** | (9,21) | **76** |
| **Sugar or sugar product** | **.** | . | **2** | (3,70) | **13** | (24,07) | **23** | (42,59) | **16** | (29,63) | **54** |
| Chocolate or chocolate product | **.** | . | **.** | . | **.** | . | **2** | (13,33) | **13** | (86,67) | **15** |
| Jam or marmalade, non-chocolate confectionery or other sugar products | **.** | . | **1** | (5,00) | **5** | (25,00) | **13** | (65,00) | **1** | (5,00) | **20** |
| Sugar, honey or syrup | **.** | . | **.** | . | **.** | . | **5** | (100,00) | **.** | . | **5** |
| Dessert and dessert sauce | **.** | . | **1** | (7,14) | **8** | (57,14) | **3** | (21,43) | **2** | (14,29) | **14** |
| **Vegetable or vegetable product** | **97** | (95,10) | **2** | (1,96) | **3** | (2,94) | **.** | . | **.** | . | **102** |
| Pulse or pulse product | **14** | (100,00) | **.** | . | **.** | . | **.** | . | **.** | . | **14** |
| Starchy root or potato | **6** | (75,00) | **1** | (12,50) | **1** | (12,50) | **.** | . | **.** | . | **8** |
| Vegetable (excluding potato) | **77** | (96,25) | **1** | (1,25) | **2** | (2,50) | **.** | . | **.** | . | **80** |
| **Beverage non-milk** | **.** | . | **6** | (17,14) | **4** | (11,43) | **.** | . | **25** | (71,43) | **35** |
| Juice or nectar | **.** | . | **2** | (33,33) | **3** | (50,00) | **.** | . | **1** | (16,67) | **6** |
| Coffee, tea, cocoa | **.** | . | **3** | (27,27) | **.** | . | **.** | . | **8** | (72,73) | **11** |
| Soft drink | **.** | . | **1** | (5,56) | **1** | (5,56) | **.** | . | **16** | (88,89) | **18** |

**Supplemental Table 4. Distribution of the main food groups and subgroups in the Nutri-Score classes in Poland (N=919 food products)**

|  | **Nutri-Score classes** | | | | | | | | | | |
| --- | --- | --- | --- | --- | --- | --- | --- | --- | --- | --- | --- |
|  | **A [Min - -1]** | | **B [0 - 2]** | | **C [3 - 10]** | | **D [11 - 18]** | | **E [19 - Max]** | | **All** |
| **Egg or egg product** | **2** | (50,00) | **.** | . | **.** | . | **1** | (25,00) | **1** | (25,00) | **4** |
| **Fat or oil** | **.** | . | **.** | . | **8** | (33,33) | **15** | (62,50) | **1** | (4,17) | **24** |
| Butter or other animal fat | **.** | . | **.** | . | **.** | . | **4** | (80,00) | **1** | (20,00) | **5** |
| Margarine or lipid of mixed origin | **.** | . | **.** | . | **3** | (37,50) | **5** | (62,50) | **.** | . | **8** |
| Vegetable fat or oil | **.** | . | **.** | . | **5** | (45,45) | **6** | (54,55) | **.** | . | **11** |
| **Fruit or fruit product** | **40** | (74,07) | **10** | (18,52) | **4** | (7,41) | **.** | . | **.** | . | **54** |
| Processed fruit product | **10** | (43,48) | **10** | (43,48) | **3** | (13,04) | **.** | . | **.** | . | **23** |
| Fresh or unprocessed fruit | **30** | (96,77) | **.** | . | **1** | (3,23) | **.** | . | **.** | . | **31** |
| **Grain or grain product** | **53** | (32,72) | **31** | (19,14) | **24** | (14,81) | **41** | (25,31) | **13** | (8,02) | **162** |
| Bread and similar products | **19** | (35,19) | **29** | (53,70) | **4** | (7,41) | **2** | (3,70) | **.** | . | **54** |
| Breakfast cereal | **3** | (27,27) | **1** | (9,09) | **3** | (27,27) | **4** | (36,36) | **.** | . | **11** |
| Cereal or cereal-like milling products and derivatives | **14** | (87,50) | **.** | . | **2** | (12,50) | **.** | . | **.** | . | **16** |
| Fine bakery ware | **.** | . | **.** | . | **15** | (23,81) | **35** | (55,56) | **13** | (20,63) | **63** |
| Pasta, rice and other cereals | **17** | (94,44) | **1** | (5,56) | **.** | . | **.** | . | **.** | . | **18** |
| **Meat or meat product** | **30** | (27,03) | **8** | (7,21) | **17** | (15,32) | **37** | (33,33) | **19** | (17,12) | **111** |
| Red meat | **8** | (38,10) | **2** | (9,52) | **5** | (23,81) | **6** | (28,57) | **.** | . | **21** |
| Poultry meat | **13** | (68,42) | **4** | (21,05) | **1** | (5,26) | **1** | (5,26) | **.** | . | **19** |
| Offal and processed meat | **9** | (12,68) | **2** | (2,82) | **11** | (15,49) | **30** | (42,25) | **19** | (26,76) | **71** |
| **Milk, milk product or milk substitute** | **12** | (18,18) | **19** | (28,79) | **11** | (16,67) | **18** | (27,27) | **6** | (9,09) | **66** |
| Cheese | **2** | (6,90) | **6** | (20,69) | **2** | (6,90) | **16** | (55,17) | **3** | (10,34) | **29** |
| Fermented milk product | **4** | (50,00) | **4** | (50,00) | **.** | . | **.** | . | **.** | . | **8** |
| Frozen dairy dessert | **.** | . | **.** | . | **2** | (100,00) | **.** | . | **.** | . | **2** |
| Milk | **6** | (22,22) | **9** | (33,33) | **7** | (25,93) | **2** | (7,41) | **3** | (11,11) | **27** |
| **Composite food product** | **102** | (44,74) | **64** | (28,07) | **27** | (11,84) | **30** | (13,16) | **5** | (2,19) | **228** |
| Meat, seafood and egg dish | **19** | (25,68) | **17** | (22,97) | **15** | (20,27) | **22** | (29,73) | **1** | (1,35) | **74** |
| Potato, pulse, vegetable and savoury cereal dish | **38** | (56,72) | **17** | (25,37) | **9** | (13,43) | **3** | (4,48) | **.** | . | **67** |
| Prepared salad | **26** | (86,67) | **3** | (10,00) | **1** | (3,33) | **.** | . | **.** | . | **30** |
| Savoury snack | **1** | (16,67) | **.** | . | **.** | . | **2** | (33,33) | **3** | (50,00) | **6** |
| Soup | **17** | (39,53) | **26** | (60,47) | **.** | . | **.** | . | **.** | . | **43** |
| Condiment or other ingredient | **1** | (12,50) | **1** | (12,50) | **2** | (25,00) | **3** | (37,50) | **1** | (12,50) | **8** |
| **Nut, seed or kernel** | **8** | (57,14) | **3** | (21,43) | **.** | . | **3** | (21,43) | **.** | . | **14** |
| Nut or seed product | **.** | . | **.** | . | **.** | . | **3** | (100,00) | **.** | . | **3** |
| Unprocessed nut, seed or kernel | **8** | (72,73) | **3** | (27,27) | **.** | . | **.** | . | **.** | . | **11** |
| **Seafood or related product** | **17** | (50,00) | **6** | (17,65) | **4** | (11,76) | **7** | (20,59) | **.** | . | **34** |
| Fish or related organism | **17** | (85,00) | **2** | (10,00) | **1** | (5,00) | **.** | . | **.** | . | **20** |
| Seafood product | **.** | . | **4** | (28,57) | **3** | (21,43) | **7** | (50,00) | **.** | . | **14** |
| **Sugar or sugar product** | **1** | (2,00) | **.** | . | **18** | (36,00) | **17** | (34,00) | **14** | (28,00) | **50** |
| Chocolate or chocolate product | **.** | . | **.** | . | **.** | . | **.** | . | **13** | (100,00) | **13** |
| Jam or marmalade, non-chocolate confectionery or other sugar products | **.** | . | **.** | . | **14** | (51,85) | **12** | (44,44) | **1** | (3,70) | **27** |
| Sugar, honey or syrup | **.** | . | **.** | . | **.** | . | **3** | (100,00) | **.** | . | **3** |
| Dessert | **1** | (14,29) | **.** | . | **4** | (57,14) | **2** | (28,57) | **.** | . | **7** |
| **Vegetable or vegetable product** | **100** | (96,15) | **3** | (2,88) | **1** | (0,96) | **.** | . | **.** | . | **104** |
| Pulse or pulse product | **16** | (100,00) | **.** | . | **.** | . | **.** | . | **.** | . | **16** |
| Starchy root or potato | **3** | (100,00) | **.** | . | **.** | . | **.** | . | **.** | . | **3** |
| Vegetable (excluding potato) | **81** | (95,29) | **3** | (3,53) | **1** | (1,18) | **.** | . | **.** | . | **85** |
| **Beverage non-milk** | **.** | . | **13** | (19,12) | **23** | (33,82) | **3** | (4,41) | **29** | (42,65) | **68** |
| Juice or nectar | **.** | . | **11** | (22,45) | **23** | (46,94) | **2** | (4,08) | **13** | (26,53) | **49** |
| Coffee, tea, cocoa | **.** | . | **2** | (50,00) | **.** | . | **.** | . | **2** | (50,00) | **4** |
| Soft drink | **.** | . | **.** | . | **.** | . | **1** | (6,67) | **14** | (93,33) | **15** |

**Supplemental Table 5. Distribution of the main food groups and subgroups in the Nutri-Score classes in Portugal (N=921 food products)**

|  | **Nutri-Score classes** | | | | | | | | | | |
| --- | --- | --- | --- | --- | --- | --- | --- | --- | --- | --- | --- |
|  | **A [Min - -1]** | | **B [0 - 2]** | | **C [3 - 10]** | | **D [11 - 18]** | | **E [19 - Max]** | | **All** |
| **Egg or egg product** | **5** | (45,45) | **1** | (9,09) | **4** | (36,36) | **1** | (9,09) | **.** | . | **11** |
| **Fat or oil** | **.** | . | **.** | . | **3** | (11,11) | **17** | (62,96) | **7** | (25,93) | **27** |
| Butter or other animal fat | **.** | . | **.** | . | **.** | . | **2** | (66,67) | **1** | (33,33) | **3** |
| Margarine or lipid of mixed origin | **.** | . | **.** | . | **3** | (18,75) | **7** | (43,75) | **6** | (37,50) | **16** |
| Vegetable fat or oil | **.** | . | **.** | . | **.** | . | **8** | (100,00) | **.** | . | **8** |
| **Fruit or fruit product** | **45** | (71,43) | **4** | (6,35) | **14** | (22,22) | **.** | . | **.** | . | **63** |
| Processed fruit product | **2** | (10,00) | **4** | (20,00) | **14** | (70,00) | **.** | . | **.** | . | **20** |
| Fresh or unprocessed fruit | **43** | (100,00) | **.** | . | **.** | . | **.** | . | **.** | . | **43** |
| **Grain or grain product** | **32** | (34,04) | **14** | (14,89) | **14** | (14,89) | **26** | (27,66) | **8** | (8,51) | **94** |
| Bread and similar products | **12** | (70,59) | **5** | (29,41) | **.** | . | **.** | . | **.** | . | **17** |
| Breakfast cereal | **3** | (27,27) | **1** | (9,09) | **5** | (45,45) | **2** | (18,18) | **.** | . | **11** |
| Cereal or cereal-like milling products and derivatives | **7** | (70,00) | **.** | . | **3** | (30,00) | **.** | . | **.** | . | **10** |
| Fine bakery ware | **.** | . | **1** | (2,86) | **2** | (5,71) | **24** | (68,57) | **8** | (22,86) | **35** |
| Pasta, rice and other cereals | **10** | (47,62) | **7** | (33,33) | **4** | (19,05) | **.** | . | **.** | . | **21** |
| **Meat or meat product** | **49** | (22,17) | **48** | (21,72) | **51** | (23,08) | **53** | (23,98) | **20** | (9,05) | **221** |
| Meat analogue | **.** | . | **.** | . | **.** | . | **1** | (50,00) | **1** | (50,00) | **2** |
| Red meat | **22** | (21,36) | **22** | (21,36) | **29** | (28,16) | **27** | (26,21) | **3** | (2,91) | **103** |
| Poultry meat | **16** | (25,81) | **22** | (35,48) | **17** | (27,42) | **7** | (11,29) | **.** | . | **62** |
| Offal and processed meat | **11** | (20,37) | **4** | (7,41) | **5** | (9,26) | **18** | (33,33) | **16** | (29,63) | **54** |
| **Milk, milk product or milk substitute** | **15** | (20,55) | **24** | (32,88) | **6** | (8,22) | **17** | (23,29) | **11** | (15,07) | **73** |
| Cheese | **2** | (8,70) | **2** | (8,70) | **2** | (8,70) | **11** | (47,83) | **6** | (26,09) | **23** |
| Fermented milk product | **5** | (33,33) | **9** | (60,00) | **1** | (6,67) | **.** | . | **.** | . | **15** |
| Frozen dairy dessert | **.** | . | **.** | . | **1** | (33,33) | **1** | (33,33) | **1** | (33,33) | **3** |
| Immitation milk products | **6** | (100,00) | **.** | . | **.** | . | **.** | . | **.** | . | **6** |
| Milk | **2** | (7,69) | **13** | (50,00) | **2** | (7,69) | **5** | (19,23) | **4** | (15,38) | **26** |
| **Composite food product** | **11** | (9,24) | **36** | (30,25) | **33** | (27,73) | **28** | (23,53) | **11** | (9,24) | **119** |
| Meat, seafood and egg dish | **8** | (12,70) | **13** | (20,63) | **25** | (39,68) | **16** | (25,40) | **1** | (1,59) | **63** |
| Potato, savoury cereal and vegetable dish | **2** | (20,00) | **3** | (30,00) | **2** | (20,00) | **2** | (20,00) | **1** | (10,00) | **10** |
| Savoury snack | **.** | . | **.** | . | **1** | (20,00) | **3** | (60,00) | **1** | (20,00) | **5** |
| Soup | **1** | (4,55) | **19** | (86,36) | **2** | (9,09) | **.** | . | **.** | . | **22** |
| Savoury sauce, condiment or other ingredient | **.** | . | **1** | (5,26) | **3** | (15,79) | **7** | (36,84) | **8** | (42,11) | **19** |
| **Nut, seed or kernel** | **7** | (50,00) | **3** | (21,43) | **3** | (21,43) | **1** | (7,14) | **.** | . | **14** |
| Nut or seed product | **2** | (50,00) | **1** | (25,00) | **.** | . | **1** | (25,00) | **.** | . | **4** |
| Unprocessed nut, seed or kernel | **5** | (50,00) | **2** | (20,00) | **3** | (30,00) | **.** | . | **.** | . | **10** |
| **Seafood or related product** | **60** | (47,62) | **40** | (31,75) | **14** | (11,11) | **12** | (9,52) | **.** | . | **126** |
| Fish or related organism | **57** | (47,50) | **38** | (31,67) | **13** | (10,83) | **12** | (10,00) | **.** | . | **120** |
| Seafood product | **3** | (50,00) | **2** | (33,33) | **1** | (16,67) | **.** | . | **.** | . | **6** |
| **Sugar or sugar product** | **.** | . | **1** | (2,44) | **13** | (31,71) | **19** | (46,34) | **8** | (19,51) | **41** |
| Chocolate or chocolate product | **.** | . | **.** | . | **1** | (14,29) | **1** | (14,29) | **5** | (71,43) | **7** |
| Jam or marmalade, non-chocolate confectionery or other sugar products | **.** | . | **.** | . | **4** | (23,53) | **11** | (64,71) | **2** | (11,76) | **17** |
| Sugar, honey or syrup | **.** | . | **.** | . | **.** | . | **3** | (100,00) | **.** | . | **3** |
| Dessert | **.** | . | **1** | (7,14) | **8** | (57,14) | **4** | (28,57) | **1** | (7,14) | **14** |
| **Vegetable or vegetable product** | **86** | (92,47) | **2** | (2,15) | **5** | (5,38) | **.** | . | **.** | . | **93** |
| Pulse or pulse product | **17** | (100,00) | **.** | . | **.** | . | **.** | . | **.** | . | **17** |
| Starchy root or potato | **5** | (50,00) | **2** | (20,00) | **3** | (30,00) | **.** | . | **.** | . | **10** |
| Vegetable (excluding potato) | **64** | (96,97) | **.** | . | **2** | (3,03) | **.** | . | **.** | . | **66** |
| **Beverage non-milk** | **5** | (12,82) | **9** | (23,08) | **7** | (17,95) | **3** | (7,69) | **15** | (38,46) | **39** |
| Juice or nectar | **.** | . | **2** | (11,76) | **6** | (35,29) | **.** | . | **9** | (52,94) | **17** |
| Coffee, tea, cocoa | **.** | . | **6** | (46,15) | **1** | (7,69) | **2** | (15,38) | **4** | (30,77) | **13** |
| Soft drink | **.** | . | **1** | (25,00) | **.** | . | **1** | (25,00) | **2** | (50,00) | **4** |
| Water | **5** | (100,00) | **.** | . | **.** | . | **.** | . | **.** | . | **5** |

**Supplemental Table 6. Distribution of the main food groups and subgroups in the Nutri-Score classes in Slovakia (N=1183 food products)**

|  | **Nutri-Score classes** | | | | | | | | | | |
| --- | --- | --- | --- | --- | --- | --- | --- | --- | --- | --- | --- |
|  | **A [Min - -1]** | | **B [0 - 2]** | | **C [3 - 10]** | | **D [11 - 18]** | | **E [19 - Max]** | | **All** |
| **Egg or egg product** | **10** | (66,67) | **1** | (6,67) | **1** | (6,67) | **3** | (20,00) | **.** | . | **15** |
| **Fat or oil** | **.** | . | **.** | . | **4** | (14,29) | **20** | (71,43) | **4** | (14,29) | **28** |
| Butter or other animal fat | **.** | . | **.** | . | **.** | . | **11** | (100,00) | **.** | . | **11** |
| Margarine or lipid of mixed origin | **.** | . | **.** | . | **1** | (50,00) | **.** | . | **1** | (50,00) | **2** |
| Vegetable fat or oil | **.** | . | **.** | . | **3** | (20,00) | **9** | (60,00) | **3** | (20,00) | **15** |
| **Fruit or fruit product** | **54** | (61,36) | **23** | (26,14) | **11** | (12,50) | **.** | . | **.** | . | **88** |
| Processed fruit product | **24** | (42,11) | **23** | (40,35) | **10** | (17,54) | **.** | . | **.** | . | **57** |
| Fresh or unprocessed fruit | **30** | (96,77) | **.** | . | **1** | (3,23) | **.** | . | **.** | . | **31** |
| **Grain or grain product** | **36** | (25,35) | **21** | (14,79) | **34** | (23,94) | **42** | (29,58) | **9** | (6,34) | **142** |
| Bread and similar products | **1** | (4,76) | **3** | (14,29) | **13** | (61,90) | **4** | (19,05) | **.** | . | **21** |
| Breakfast cereal | **2** | (6,90) | **.** | . | **3** | (10,34) | **18** | (62,07) | **6** | (20,69) | **29** |
| Cereal or cereal-like milling products and derivatives | **14** | (48,28) | **7** | (24,14) | **7** | (24,14) | **1** | (3,45) | **.** | . | **29** |
| Fine bakery ware | **.** | . | **3** | (9,09) | **9** | (27,27) | **18** | (54,55) | **3** | (9,09) | **33** |
| Pasta, rice and other cereals | **19** | (63,33) | **8** | (26,67) | **2** | (6,67) | **1** | (3,33) | **.** | . | **30** |
| **Meat or meat product** | **107** | (56,32) | **28** | (14,74) | **16** | (8,42) | **38** | (20,00) | **1** | (0,53) | **190** |
| Red meat | **29** | (64,44) | **4** | (8,89) | **7** | (15,56) | **5** | (11,11) | **.** | . | **45** |
| Poultry meat | **17** | (60,71) | **2** | (7,14) | **2** | (7,14) | **7** | (25,00) | **.** | . | **28** |
| Offal and processed meat | **61** | (52,14) | **22** | (18,80) | **7** | (5,98) | **26** | (22,22) | **1** | (0,85) | **117** |
| **Milk, milk product or milk substitute** | **22** | (37,93) | **15** | (25,86) | **13** | (22,41) | **3** | (5,17) | **5** | (8,62) | **58** |
| Cheese | **7** | (33,33) | **2** | (9,52) | **9** | (42,86) | **1** | (4,76) | **2** | (9,52) | **21** |
| Fermented milk product | **7** | (53,85) | **2** | (15,38) | **4** | (30,77) | **.** | . | **.** | . | **13** |
| Immitation milk products | **2** | (100,00) | **.** | . | **.** | . | **.** | . | **.** | . | **2** |
| Milk | **6** | (27,27) | **11** | (50,00) | **.** | . | **2** | (9,09) | **3** | (13,64) | **22** |
| **Composite food product** | **10** | (3,56) | **74** | (26,33) | **107** | (38,08) | **62** | (22,06) | **28** | (9,96) | **281** |
| Meat, seafood and egg dish | **1** | (1,32) | **15** | (19,74) | **22** | (28,95) | **20** | (26,32) | **18** | (23,68) | **76** |
| Potato, pulse, vegetable and savoury cereal dish | **8** | (5,84) | **31** | (22,63) | **53** | (38,69) | **36** | (26,28) | **9** | (6,57) | **137** |
| Prepared salad | **.** | . | **.** | . | **2** | (100,00) | **.** | . | **.** | . | **2** |
| Savoury snack | **.** | . | **.** | . | **1** | (16,67) | **4** | (66,67) | **1** | (16,67) | **6** |
| Soup | **1** | (2,22) | **27** | (60,00) | **17** | (37,78) | **.** | . | **.** | . | **45** |
| Savoury sauce, condiment or other ingredient | **.** | . | **1** | (6,67) | **12** | (80,00) | **2** | (13,33) | **.** | . | **15** |
| **Nut, seed or kernel** | **9** | (28,13) | **7** | (21,88) | **13** | (40,63) | **3** | (9,38) | **.** | . | **32** |
| Nut or seed product | **1** | (25,00) | **.** | . | **1** | (25,00) | **2** | (50,00) | **.** | . | **4** |
| Unprocessed nut, seed or kernel | **8** | (28,57) | **7** | (25,00) | **12** | (42,86) | **1** | (3,57) | **.** | . | **28** |
| **Seafood or related product** | **49** | (62,03) | **17** | (21,52) | **5** | (6,33) | **8** | (10,13) | **.** | . | **79** |
| Fish or related organism | **42** | (84,00) | **7** | (14,00) | **.** | . | **1** | (2,00) | **.** | . | **50** |
| Seafood product | **7** | (24,14) | **10** | (34,48) | **5** | (17,24) | **7** | (24,14) | **.** | . | **29** |
| **Sugar or sugar product** | **.** | . | **2** | (2,78) | **10** | (13,89) | **57** | (79,17) | **3** | (4,17) | **72** |
| Chocolate or chocolate product | **.** | . | **.** | . | **.** | . | **15** | (88,24) | **2** | (11,76) | **17** |
| Jam or marmalade, non-chocolate confectionery or other sugar products | **.** | . | **.** | . | **4** | (10,26) | **35** | (89,74) | **.** | . | **39** |
| Sugar, honey or syrup | **.** | . | **.** | . | **.** | . | **5** | (100,00) | **.** | . | **5** |
| Dessert | **.** | . | **2** | (18,18) | **6** | (54,55) | **2** | (18,18) | **1** | (9,09) | **11** |
| **Vegetable or vegetable product** | **109** | (88,62) | **10** | (8,13) | **4** | (3,25) | **.** | . | **.** | . | **123** |
| Pulse or pulse product | **15** | (100,00) | **.** | . | **.** | . | **.** | . | **.** | . | **15** |
| Starchy root or potato | **1** | (20,00) | **4** | (80,00) | **.** | . | **.** | . | **.** | . | **5** |
| Vegetable (excluding potato) | **93** | (90,29) | **6** | (5,83) | **4** | (3,88) | **.** | . | **.** | . | **103** |
| **Beverage non-milk** | **18** | (24,00) | **3** | (4,00) | **15** | (20,00) | **8** | (10,67) | **31** | (41,33) | **75** |
| Juice or nectar | **.** | . | **3** | (15,79) | **13** | (68,42) | **2** | (10,53) | **1** | (5,26) | **19** |
| Coffee, tea, cocoa | **.** | . | **.** | . | **.** | . | **2** | (25,00) | **6** | (75,00) | **8** |
| Soft drink | **.** | . | **.** | . | **2** | (6,67) | **4** | (13,33) | **24** | (80,00) | **30** |
| Water | **18** | (100,00) | **.** | . | **.** | . | **.** | . | **.** | . | **18** |

**Supplemental Table 7. Distribution of the main food groups and subgroups in the Nutri-Score classes in Sweden (N=1990 food products)**

|  | **Nutri-Score classes** | | | | | | | | | | |
| --- | --- | --- | --- | --- | --- | --- | --- | --- | --- | --- | --- |
|  | **A [Min - -1]** | | **B [0 - 2]** | | **C [3 - 10]** | | **D [11 - 18]** | | **E [19 - Max]** | | **All** |
| **Egg or egg product** | **5** | (55,56) | **1** | (11,11) | **1** | (11,11) | **2** | (22,22) | **.** | . | **9** |
| **Fat or oil** | **.** | . | **.** | . | **10** | (15,38) | **43** | (66,15) | **12** | (18,46) | **65** |
| Butter or other animal fat | **.** | . | **.** | . | **1** | (14,29) | **3** | (42,86) | **3** | (42,86) | **7** |
| Margarine or lipid of mixed origin | **.** | . | **.** | . | **4** | (9,52) | **30** | (71,43) | **8** | (19,05) | **42** |
| Vegetable fat or oil | **.** | . | **.** | . | **5** | (31,25) | **10** | (62,50) | **1** | (6,25) | **16** |
| **Fruit or fruit product** | **63** | (67,74) | **20** | (21,51) | **10** | (10,75) | **.** | . | **.** | . | **93** |
| Processed fruit product | **11** | (26,83) | **20** | (48,78) | **10** | (24,39) | **.** | . | **.** | . | **41** |
| Fresh or unprocessed fruit | **52** | (100,00) | **.** | . | **.** | . | **.** | . | **.** | . | **52** |
| **Grain or grain product** | **113** | (37,29) | **48** | (15,84) | **52** | (17,16) | **58** | (19,14) | **32** | (10,56) | **303** |
| Bread and similar products | **40** | (49,38) | **18** | (22,22) | **19** | (23,46) | **2** | (2,47) | **2** | (2,47) | **81** |
| Breakfast cereal and cereal bar | **10** | (30,30) | **2** | (6,06) | **10** | (30,30) | **11** | (33,33) | **.** | . | **33** |
| Cereal or cereal-like milling products and derivatives | **15** | (57,69) | **2** | (7,69) | **3** | (11,54) | **4** | (15,38) | **2** | (7,69) | **26** |
| Fine bakery ware | **.** | . | **3** | (3,90) | **8** | (10,39) | **38** | (49,35) | **28** | (36,36) | **77** |
| Pasta, rice and other cereals | **48** | (55,81) | **23** | (26,74) | **12** | (13,95) | **3** | (3,49) | **.** | . | **86** |
| **Meat or meat product** | **101** | (33,89) | **40** | (13,42) | **37** | (12,42) | **75** | (25,17) | **45** | (15,10) | **298** |
| Meat analogue | **3** | (75,00) | **.** | . | **.** | . | **1** | (25,00) | **.** | . | **4** |
| Red meat | **55** | (49,55) | **22** | (19,82) | **15** | (13,51) | **15** | (13,51) | **4** | (3,60) | **111** |
| Poultry meat | **20** | (47,62) | **7** | (16,67) | **5** | (11,90) | **9** | (21,43) | **1** | (2,38) | **42** |
| Offal and processed meat | **23** | (16,31) | **11** | (7,80) | **17** | (12,06) | **50** | (35,46) | **40** | (28,37) | **141** |
| **Milk, milk product or milk substitute** | **19** | (13,38) | **25** | (17,61) | **36** | (25,35) | **53** | (37,32) | **9** | (6,34) | **142** |
| Cheese | **2** | (3,70) | **7** | (12,96) | **11** | (20,37) | **29** | (53,70) | **5** | (9,26) | **54** |
| Fermented milk product | **11** | (40,74) | **6** | (22,22) | **8** | (29,63) | **1** | (3,70) | **1** | (3,70) | **27** |
| Frozen dairy dessert | **.** | . | **1** | (5,00) | **8** | (40,00) | **11** | (55,00) | **.** | . | **20** |
| Immitation milk products | **1** | (7,69) | **4** | (30,77) | **4** | (30,77) | **3** | (23,08) | **1** | (7,69) | **13** |
| Milk | **5** | (17,86) | **7** | (25,00) | **5** | (17,86) | **9** | (32,14) | **2** | (7,14) | **28** |
| **Composite food product** | **97** | (18,06) | **155** | (28,86) | **157** | (29,24) | **110** | (20,48) | **18** | (3,35) | **537** |
| Meat, seafood and egg dish | **29** | (14,01) | **67** | (32,37) | **55** | (26,57) | **50** | (24,15) | **6** | (2,90) | **207** |
| Potato, pulse, vegetable and savoury cereal dish | **53** | (31,93) | **54** | (32,53) | **39** | (23,49) | **20** | (12,05) | **.** | . | **166** |
| Prepared salad | **6** | (46,15) | **3** | (23,08) | **3** | (23,08) | **1** | (7,69) | **.** | . | **13** |
| Savoury snack | **1** | (8,33) | **1** | (8,33) | **.** | . | **7** | (58,33) | **3** | (25,00) | **12** |
| Soup | **7** | (14,00) | **23** | (46,00) | **20** | (40,00) | **.** | . | **.** | . | **50** |
| Savoury sauce, condiment or other ingredient | **1** | (1,12) | **7** | (7,87) | **40** | (44,94) | **32** | (35,96) | **9** | (10,11) | **89** |
| **Nut, seed or kernel** | **7** | (28,00) | **.** | . | **15** | (60,00) | **3** | (12,00) | **.** | . | **25** |
| Nut or seed product | **3** | (15,79) | **.** | . | **13** | (68,42) | **3** | (15,79) | **.** | . | **19** |
| Unprocessed nut, seed or kernel | **4** | (66,67) | **.** | . | **2** | (33,33) | **.** | . | **.** | . | **6** |
| **Seafood or related product** | **51** | (43,22) | **15** | (12,71) | **10** | (8,47) | **38** | (32,20) | **4** | (3,39) | **118** |
| Fish or related organism | **46** | (77,97) | **5** | (8,47) | **2** | (3,39) | **6** | (10,17) | **.** | . | **59** |
| Seafood product | **5** | (8,47) | **10** | (16,95) | **8** | (13,56) | **32** | (54,24) | **4** | (6,78) | **59** |
| **Sugar or sugar product** | **3** | (2,50) | **11** | (9,17) | **38** | (31,67) | **42** | (35,00) | **26** | (21,67) | **120** |
| Chocolate or chocolate product | **.** | . | **.** | . | **1** | (3,57) | **5** | (17,86) | **22** | (78,57) | **28** |
| Jam or marmalade, non-chocolate confectionery or other sugar products | **1** | (2,70) | **1** | (2,70) | **13** | (35,14) | **20** | (54,05) | **2** | (5,41) | **37** |
| Sugar, honey or syrup | **.** | . | **.** | . | **1** | (14,29) | **6** | (85,71) | **.** | . | **7** |
| Dessert and dessert sauce | **2** | (4,17) | **10** | (20,83) | **23** | (47,92) | **11** | (22,92) | **2** | (4,17) | **48** |
| **Vegetable or vegetable product** | **189** | (90,87) | **8** | (3,85) | **10** | (4,81) | **1** | (0,48) | **.** | . | **208** |
| Pulse or pulse product | **32** | (96,97) | **1** | (3,03) | **.** | . | **.** | . | **.** | . | **33** |
| Starchy root or potato | **22** | (88,00) | **2** | (8,00) | **1** | (4,00) | **.** | . | **.** | . | **25** |
| Vegetable (excluding potato) | **135** | (90,00) | **5** | (3,33) | **9** | (6,00) | **1** | (0,67) | **.** | . | **150** |
| **Beverage non-milk** | **5** | (6,94) | **19** | (26,39) | **4** | (5,56) | **11** | (15,28) | **33** | (45,83) | **72** |
| Juice or nectar | **.** | . | **11** | (55,00) | **2** | (10,00) | **4** | (20,00) | **3** | (15,00) | **20** |
| Coffee, tea, cocoa | **.** | . | **6** | (40,00) | **1** | (6,67) | **1** | (6,67) | **7** | (46,67) | **15** |
| Soft drink | **.** | . | **2** | (6,25) | **1** | (3,13) | **6** | (18,75) | **23** | (71,88) | **32** |
| Water | **5** | (100,00) | **.** | . | **.** | . | **.** | . | **.** | . | **5** |

**Supplemental Table 8. Distribution of the main food groups and subgroups in the Nutri-Score classes in Switzerland (N=849 food products)**

|  | **Nutri-Score classes** | | | | | | | | | | |
| --- | --- | --- | --- | --- | --- | --- | --- | --- | --- | --- | --- |
|  | **A [Min - -1]** | | **B [0 - 2]** | | **C [3 - 10]** | | **D [11 - 18]** | | **E [19 - Max]** | | **All** |
| **Egg or egg product** | **2** | (66,67) | **.** | . | **.** | . | **1** | (33,33) | **.** | . | **3** |
| **Fat or oil** | **.** | . | **.** | . | **9** | (33,33) | **16** | (59,26) | **2** | (7,41) | **27** |
| Butter or other animal fat | **.** | . | **.** | . | **.** | . | **5** | (83,33) | **1** | (16,67) | **6** |
| Margarine or lipid of mixed origin | **.** | . | **.** | . | **7** | (70,00) | **3** | (30,00) | **.** | . | **10** |
| Vegetable fat or oil | **.** | . | **.** | . | **2** | (18,18) | **8** | (72,73) | **1** | (9,09) | **11** |
| **Fruit or fruit product** | **41** | (70,69) | **12** | (20,69) | **5** | (8,62) | **.** | . | **.** | . | **58** |
| Processed fruit product | **8** | (34,78) | **12** | (52,17) | **3** | (13,04) | **.** | . | **.** | . | **23** |
| Fresh or unprocessed fruit | **33** | (94,29) | **.** | . | **2** | (5,71) | **.** | . | **.** | . | **35** |
| **Grain or grain product** | **57** | (29,38) | **18** | (9,28) | **33** | (17,01) | **54** | (27,84) | **32** | (16,49) | **194** |
| Bread and similar products | **7** | (25,93) | **13** | (48,15) | **7** | (25,93) | **.** | . | **.** | . | **27** |
| Breakfast cereal and cereal bar | **11** | (25,58) | **1** | (2,33) | **11** | (25,58) | **13** | (30,23) | **7** | (16,28) | **43** |
| Cereal or cereal-like milling products and derivatives | **11** | (78,57) | **.** | . | **3** | (21,43) | **.** | . | **.** | . | **14** |
| Fine bakery ware | **.** | . | **2** | (2,50) | **12** | (15,00) | **41** | (51,25) | **25** | (31,25) | **80** |
| Pasta, rice and other cereals | **28** | (93,33) | **2** | (6,67) | **.** | . | **.** | . | **.** | . | **30** |
| **Meat or meat product** | **59** | (46,46) | **16** | (12,60) | **6** | (4,72) | **16** | (12,60) | **30** | (23,62) | **127** |
| Red meat | **42** | (65,63) | **11** | (17,19) | **5** | (7,81) | **5** | (7,81) | **1** | (1,56) | **64** |
| Poultry meat | **11** | (78,57) | **2** | (14,29) | **.** | . | **1** | (7,14) | **.** | . | **14** |
| Offal and processed meat | **6** | (12,24) | **3** | (6,12) | **1** | (2,04) | **10** | (20,41) | **29** | (59,18) | **49** |
| **Milk, milk product or milk substitute** | **18** | (16,07) | **22** | (19,64) | **15** | (13,39) | **49** | (43,75) | **8** | (7,14) | **112** |
| Cheese | **5** | (10,00) | **4** | (8,00) | **7** | (14,00) | **29** | (58,00) | **5** | (10,00) | **50** |
| Fermented milk product | **5** | (38,46) | **6** | (46,15) | **2** | (15,38) | **.** | . | **.** | . | **13** |
| Frozen dairy dessert | **4** | (19,05) | **.** | . | **4** | (19,05) | **13** | (61,90) | **.** | . | **21** |
| Immitation milk products | **1** | (50,00) | **1** | (50,00) | **.** | . | **.** | . | **.** | . | **2** |
| Milk | **3** | (11,54) | **11** | (42,31) | **2** | (7,69) | **7** | (26,92) | **3** | (11,54) | **26** |
| **Composite food product** | **5** | (5,05) | **28** | (28,28) | **47** | (47,47) | **14** | (14,14) | **5** | (5,05) | **99** |
| Meat and seafood dish | **1** | (20,00) | **2** | (40,00) | **.** | . | **1** | (20,00) | **1** | (20,00) | **5** |
| Potato, vegetable and savoury cereal dish | **1** | (4,17) | **11** | (45,83) | **11** | (45,83) | **1** | (4,17) | **.** | . | **24** |
| Prepared salad | **.** | . | **2** | (66,67) | **1** | (33,33) | **.** | . | **.** | . | **3** |
| Sandwich | **1** | (100,00) | **.** | . | **.** | . | **.** | . | **.** | . | **1** |
| Savoury snack | **.** | . | **.** | . | **5** | (45,45) | **6** | (54,55) | **.** | . | **11** |
| Soup | **.** | . | **7** | (25,93) | **20** | (74,07) | **.** | . | **.** | . | **27** |
| Savoury sauce, condiment or other ingredient | **2** | (7,14) | **6** | (21,43) | **10** | (35,71) | **6** | (21,43) | **4** | (14,29) | **28** |
| **Nut, seed or kernel** | **6** | (31,58) | **5** | (26,32) | **4** | (21,05) | **3** | (15,79) | **1** | (5,26) | **19** |
| Nut or seed product | **.** | . | **1** | (14,29) | **2** | (28,57) | **3** | (42,86) | **1** | (14,29) | **7** |
| Unprocessed nut, seed or kernel | **6** | (50,00) | **4** | (33,33) | **2** | (16,67) | **.** | . | **.** | . | **12** |
| **Seafood or related product** | **21** | (65,63) | **5** | (15,63) | **2** | (6,25) | **3** | (9,38) | **1** | (3,13) | **32** |
| Fish or related organism | **19** | (90,48) | **2** | (9,52) | **.** | . | **.** | . | **.** | . | **21** |
| Seafood product | **2** | (18,18) | **3** | (27,27) | **2** | (18,18) | **3** | (27,27) | **1** | (9,09) | **11** |
| **Sugar or sugar product** | **.** | . | **1** | (2,56) | **10** | (25,64) | **12** | (30,77) | **16** | (41,03) | **39** |
| Chocolate or chocolate product | **.** | . | **.** | . | **1** | (5,56) | **2** | (11,11) | **15** | (83,33) | **18** |
| Jam or marmalade, non-chocolate confectionery or other sugar products | **.** | . | **.** | . | **3** | (33,33) | **5** | (55,56) | **1** | (11,11) | **9** |
| Sugar, honey or syrup | **.** | . | **.** | . | **.** | . | **5** | (100,00) | **.** | . | **5** |
| Dessert and dessert sauce | **.** | . | **1** | (14,29) | **6** | (85,71) | **.** | . | **.** | . | **7** |
| **Vegetable or vegetable product** | **65** | (98,48) | **1** | (1,52) | **.** | . | **.** | . | **.** | . | **66** |
| Pulse or pulse product | **11** | (100,00) | **.** | . | **.** | . | **.** | . | **.** | . | **11** |
| Starchy root or potato | **3** | (75,00) | **1** | (25,00) | **.** | . | **.** | . | **.** | . | **4** |
| Vegetable (excluding potato) | **51** | (100,00) | **.** | . | **.** | . | **.** | . | **.** | . | **51** |
| **Beverage non-milk** | **27** | (36,99) | **7** | (9,59) | **15** | (20,55) | **7** | (9,59) | **17** | (23,29) | **73** |
| Juice or nectar | **.** | . | **3** | (21,43) | **8** | (57,14) | **2** | (14,29) | **1** | (7,14) | **14** |
| Coffee, tea, cocoa | **.** | . | **1** | (16,67) | **2** | (33,33) | **.** | . | **3** | (50,00) | **6** |
| Soft drink | **.** | . | **3** | (11,54) | **5** | (19,23) | **5** | (19,23) | **13** | (50,00) | **26** |
| Water | **27** | (100,00) | **.** | . | **.** | . | **.** | . | **.** | . | **27** |
